# Psychometric validation and testing of the 10-item pediatric daily chest-related electronic patient reported outcome (ePRO) diary

**DOI:** 10.1186/s41687-023-00546-2

**Published:** 2023-01-25

**Authors:** Rob Arbuckle, Tim Shea, Kate Burrows, Chris Marshall, Andrew Trigg, Julia Stein, Helmut H. Albrecht

**Affiliations:** 1 Adelphi Values Patient-Centered Outcomes, Bollington, UK; 2 RB Health (US), LLC, Parsippany, NJ USA; 3 Adelphi Values Patient-Centered Outcomes (at the time research was conducted), Bollington, UK; 4 Herbert Wertheim College of Medicine, Cellular Biology and Pharmacology, Florida International University, Miami, FL USA

**Keywords:** Patient-reported outcome development, Pediatrics, Psychometric validation, ePRO diary, Exit interviews

## Abstract

**Background and objective:**

The chest-related electronic patient reported outcome (ePRO) diary was recently developed to assess chest-related symptoms experienced by pediatric and adolescent populations during upper respiratory tract infections (URTI). The objective of this research was the psychometric evaluation of the chest-related ePRO diary in pediatric, adolescent and adult participants.

**Methods:**

This non-interventional, psychometric validation study involved participants (N = 195; n = 42 6–8 years; n = 47 9–11 years; n = 55 12–17 years, n = 51 18+ years) completing the chest-related ePRO diary twice daily for 10 days while experiencing an acute URTI. Preliminary item-level performance and dimensionality results, along with consideration of previous qualitative findings, were used to inform item reduction decisions, the structure of the measure and scoring algorithm development. Subsequent analyses on the finalized measure included assessments of reliability (internal consistency and test-retest reliability), construct validity (convergent validity and known groups validity) and ability to detect change. Comparisons of findings were made between the different age groups as part of the analyses to assess the psychometric properties of the chest-related ePRO diary and to characterize potential differences in the symptom experience of children, adolescents, and adults.

**Results:**

The measure demonstrated strong quality of completion and showed relatively similar trajectories of symptom scores over time within different age subgroups and good item response distribution properties. Exploratory factor analysis supported a one-factor solution in the total population and within age subgroups, and test-retest reliability of the measure was strong (Intra-class correlation: 0.843–0.894 between Visit 1 and Day 1). The measure also demonstrated strong construct validity through high correlations with relevant items on the Child Cold Symptom Questionnaire (CCSQ), strong known groups validity (with statistically significant differences between severity groups) and was responsive to change over time with change groups defined based on change on global items.

**Conclusion:**

The findings demonstrate that the chest-related ePRO diary provides a valid, reliable, responsive measure of chest congestion symptoms experienced with the common cold in pediatric and adolescent populations, and that only minor differences are present in the disease trajectory when comparing adults to younger participants, supporting the use of the measure in interventional studies.

**Supplementary Information:**

The online version contains supplementary material available at 10.1186/s41687-023-00546-2.

## Background

The common cold, an upper respiratory tract infection (URTI), is the most common acute illness in the United States (US), affecting both pediatric and adult populations, and resulting in more doctor visits and disruption to employment and education than any other illness. Medications for cough and cold symptoms are readily available over-the-counter (OTC) and are “Generally Recognized as Safe and Effective” (GRASE) under the OTC monograph system (Title 21 of the *Code of Federal Regulations*, Part 341). However, evidence of efficacy in children generated through randomized controlled trials is lacking. This is likely due in part to difficulties in evaluating common cold symptom severity and changes over time in adolescent and pediatric populations [1].

The newly developed 10-item pediatric daily chest-related electronic patient reported outcome (ePRO) diary is designed to evaluate the symptoms of chest congestion experienced as part of the common cold. The pediatric ePRO diary was developed through extensive, rigorous qualitative research in a sample of 49 participants, including 27 children aged 6–11 years old and 12 adolescents aged 12–17, who had current or recent colds with chest congestion symptoms. The qualitative research also included testing of the items with a sample of 10 adults, aged 18 and older, who had a common cold with chest symptoms. Results of the qualitative research demonstrated a high degree of consistency across age groups in terms of item relevance, and with best performing items well understood in all age groups. Each participant took part in concept elicitation (CE) interviews exploring their experience of chest congestion and related symptoms, followed by 2–5 days of at-home completion of draft patient PRO items on a hand-held electronic device and a cognitive debriefing (CD) interview. Cognitive debriefing was performed on the electronic version of the PRO and included exploration of usability and feasibility of completing the diary on a hand-held electronic device.

The aim of the present study was to evaluate the psychometric properties of the novel 10-item chest congestion PRO measure, especially focusing on reliability and validity in a pediatric population and feasibility of children completing ePRO assessments twice daily without parent or caregiver assistance. A secondary objective was to explore any similarities and differences in the experience of chest-related symptoms as experienced by children, adolescents, and adults.

## Methods

### Study design

**Fig. 1:**
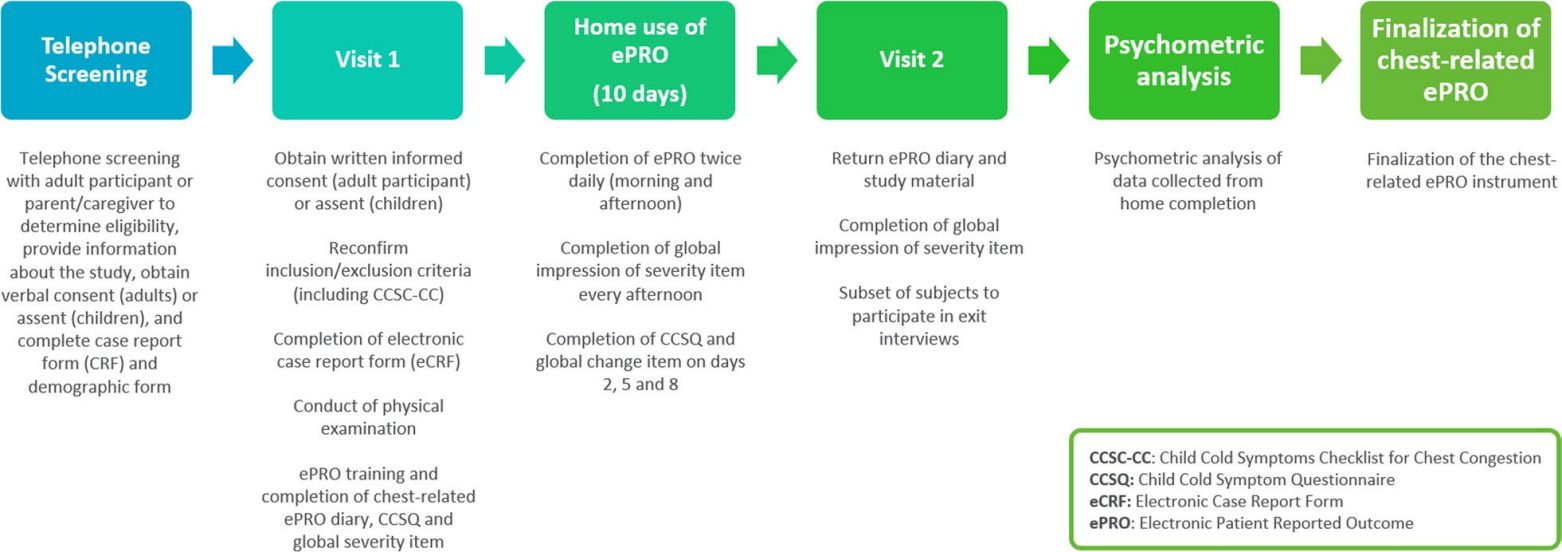
Overview of study methodology

This study was a multi-centre, observational, non-interventional study, in which PRO data were collected directly from participants using an ePRO diary, with a subset of participants also taking part in an exit interview. Study participants were not required to adhere to or refrain from any specific treatment of their URTI during the course of the study. Initial analyses of item performance and dimensionality were performed to inform item reduction and scoring algorithm development, followed by assessments of reliability, construct validity and ability to detect change over time of the resulting scores. Key differences between age groups were also evaluated. An overview of the study is provided in Fig. 1.

### Sample and recruitment

A total sample of approximately 200 participants, divided evenly across four specific age groups, was targeted for inclusion in the study: 6–8-year-olds (n = 50); 9–11-year-olds (n = 50); 12–17-year-olds (n = 50) and 18 + year olds (n = 50). For psychometric analyses, a ratio of 10 participants per instrument item is often used as a ‘rule of thumb’ for determining an adequate sample size [2]. With the chest-related ePRO diary consisting of 10 morning and evening items (20 total) the target sample of 200 participants was deemed sufficient. The narrow age bands for child and adolescent participants were devised in line with existing research and industry guidance that PRO evaluation in pediatric populations should be performed within narrow age bands to ensure items/scores perform adequately across samples that vary in terms of age/developmentally [3, 4]. Target quotas were also used to ensure good representation of both male and female participants, and participants with diverse ethnic backgrounds, thus ensuring the generalisability of the results.

The sample was recruited from 13 clinical sites in the US. Potential participants were identified through advertizing and contacting adults and parent/caregivers of children on databases at the study sites, soliciting volunteers to participate in the study if they/their child had a current cold. A telephone screening was performed prior to Study Visit 1 to reduce the rate of screen failures (i.e., participants who did not meet the study eligibility criteria). Eligible participants experiencing symptoms associated with an acute URTI, were then enrolled into the study by the recruiting clinician within 72 h of onset of cold symptoms. Participants were eligible if they responded at least ’some’ or ‘hard’ for one of the three chest congestion symptoms and at least ‘some’ for one of the other cold symptoms present on the Child Cold Symptoms Checklist for Chest Congestion (CCSC-CC) [5] at the time of the initial telephone screening and at Visit 1. The CCSC-CC was developed based on the CCSQ during the development of the CCSQ for the purpose of identifying/screening children with the common cold for inclusion in a research study.

### Study assessments

**Fig. 2:**
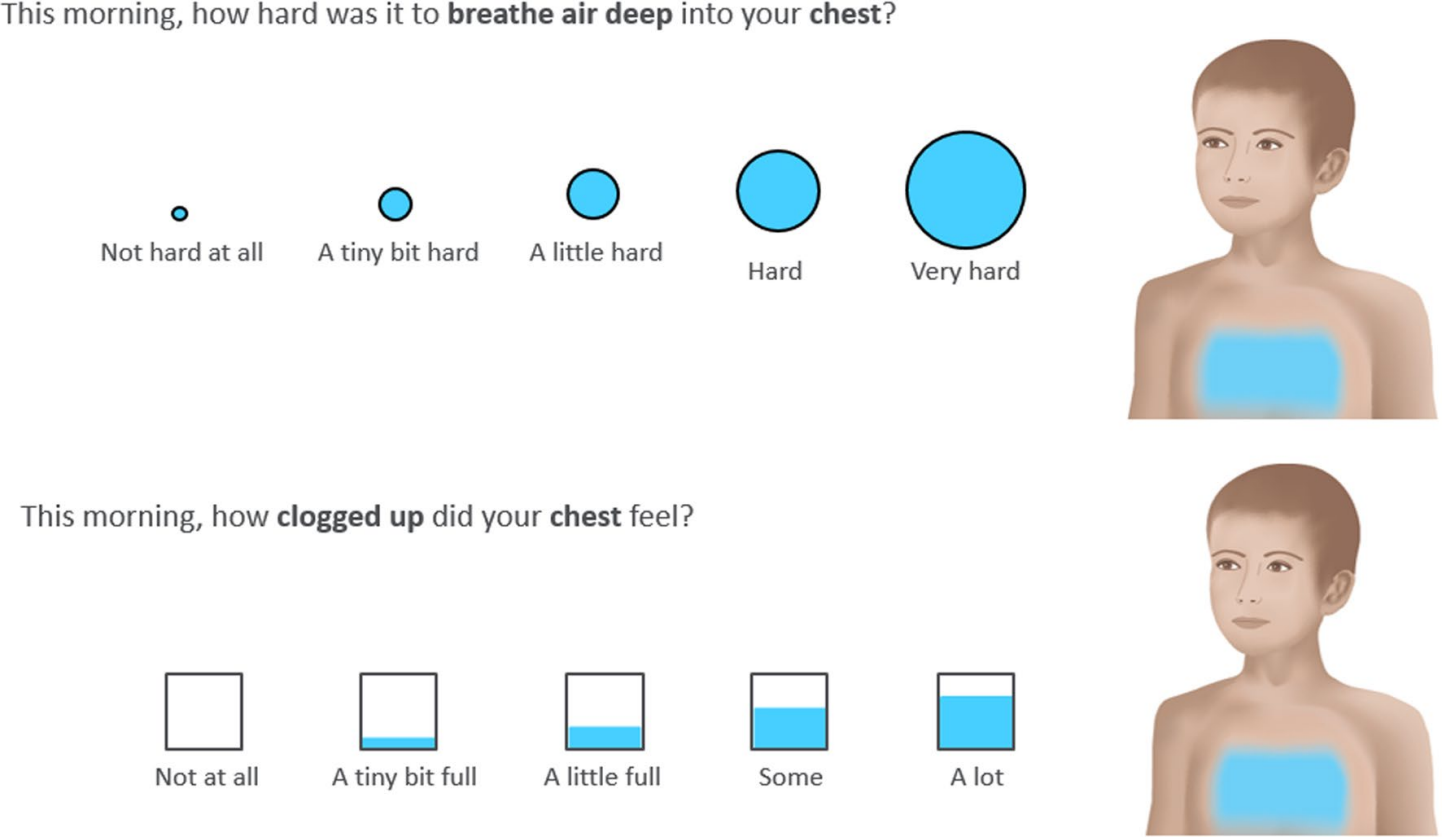
Chest related ePRO example items and response options

The newly developed chest-related ePRO diary consisted of ten items designed to evaluate the symptoms of chest congestion experienced as part of the common cold or a URTI. The measure has been designed for twice daily assessment; the content of the morning and afternoon items are consistent with each other, but with a recall period specific to the completion time. Item responses were recorded on a 5-point verbal descriptor scale (scored from 0 to 4), with each response option illustrated pictorially with circles or boxes of increasing size and volume. Interpretation aids were also provided in the form of an illustration of a gender-neutral, emotionless child with the body area of the symptom assessed shaded in blue (Fig. 2). The chest-related ePRO diary was completed twice daily during the ten day at-home completion period [6].

The 10 items assessed difficulty breathing (1 item), chest tightness (1 item), chest pain (1 item), chest feeling heavy (1 item), chest feeling full of mucus (goo)/stuffed up/clogged up (3 items) and difficulty clearing/coughing up mucus (goo) from chest/throat (3 items).

Other PRO measures were administered during the study for screening purposes and to aid validation of the chest-related ePRO as detailed in Table 1.

**Table 1:** PRO measures and checklists administered during the study to aid validation of the chest-related ePRO

Measure/checklist	Description and response scale	Time points for completion
Child Cold Symptom Questionnaire (CCSQ)	Child completed PRO consisting of 32 items (15 morning; 17 afternoon) Evidence of validity as a measure of cold symptom in children has been previously confirmed [5] Items all answered on a five-point verbal descriptor scale (scored from 0 to 4)	Completed by all participants (including adults) on the mornings and afternoons of Days 2, 5 and 8
Child Cold Symptom Checklist for Chest Congestion (CCSC-CC)	9-item checklist assessing chest congestion symptoms Responses on the CCSC-CC were recorded on a five-point verbal descriptor scale. Developed as a screening tool during previous research in a pediatric common cold population [5]	Administered at screening to ensure participants were experiencing an adequate number and severity of cold symptoms for inclusion in the study. Also included to assess child participants’ reading ability
Child Global Impression of Severity (CGI-S)	Single-item global assessment asking “How bad is your cold today?” Answers recorded on a 0–4 verbal descriptor response scale (‘no cold’, ‘a tiny bit bad’, ‘a little bad’, ‘bad’ and ‘very bad’). The CGI-S item was drafted in line with common practice for static global impression items assessing disease severity [7, 8]. Further, the descriptor words used for the CGI-S response scale (e.g., ‘a tiny bit’ and a ‘little bit’) were similar to those included in the chest-related ePRO itself, where cognitive debriefing (CD) evidence suggested good understanding of response scales [6]	The CGI-S was completed by all participants at Visit 1 and each afternoon during the ten day at-home completion period
Child Global Impression of Change (CGI-C)	The CGI-C was a single global item which asks “How much has your cold changed since Visit 1 when you started the study? Response options: ‘much better’, ‘a little better’, ‘the same’, ‘a little worse’, ‘much worse’. Similar to the CGI-S, the CGI-C item was drafted in line with common practice for impression of change items, and ensuring appropriate wording for pediatric completion [7, 8]	The CGI-S was completed by all participants at Visit 1 and each afternoon during the ten day at-home completion period

### Psychometric analyses

Analyses of the chest-related ePRO diary began with an evaluation of the distributional properties of the individual items. Further, the factorial structure of the ePRO items was explored to inform item reduction and scoring decisions. Following identification of final items, the following properties were evaluated: internal consistency and test-retest reliability, concurrent validity, known groups validity and ability to detect change. Where possible, psychometric analyses were conducted in four narrow age-groups (6–8 year-olds, 9–11 year-olds, 12–17 year-olds and adults) but for analyses requiring larger sample sizes, age groups were collapsed.

To adjust for multiplicity in item response theory (IRT) and differential item functioning (DIF) analyses, the threshold of statistical significance was set to p < 0.01. Otherwise no adjustments were made for multiplicity, as is typical in psychometric evaluation. All analyses apart from exploratory factor analysis (EFA) and IRT were conducted using SAS^®^ version 9.4 according to a pre-developed statistical analysis plan. EFA was conducted using Mplus (Version 8) [9] and IRT was conducted in IRT PRO (Version 3) [10]. Details of all psychometric analyses are provided below in Table 2.

**Table 2:** Description and criteria for performed psychometric analyses

Property	Description	Criteria for consideration
Quality of completion	Evaluated frequency and percentage of item-level missing data per participant; number and percentage of participants with at least one missing completion; number and percentage of participants with at least one missing item per completion; number and percentage of participants with no missing data; number and percentage of missing data for each item at each morning and afternoon completion	Issues with completion/time points provide information regarding feasibility of completing an ePRO diary by children twice daily without caregiver assistance
Item response distributions and item performance	Item response distributions were examined and percentages of minimum and maximum responses were calculated to examine floor and ceiling effects for all of the chest ePRO items, and mean scores for each individual chest-related ePRO diary item were evaluated for each time point collected (morning and evening, Day 1-Day 10) and between age groups Inter-item correlations were examined to gauge the strength of the relationships among items and the appropriateness of scoring them together	For inter-item correlations, moderate to high correlations (0.40–0.80) were expected and items with high (Pearson correlation r > 0.80) were flagged for further consideration
Differential item functioning (DIF)	DIF analyses were conducted using ordinal logistic regression models, to investigate whether the different chest-related ePRO diary items performed in the same manner across age groups	If the coefficient of age was significant (p < 0.01), this was considered evidence of uniform DIF. If interaction coefficient between age group and CGI-S was significant (p < 0.01), considered evidence of non-uniform DIF
Exploratory factor analysis (EFA)	EFA was conducted to explore the potential item-scale structure for the chest-related ePRO diary before item deletions. EFA was used to explore factor solutions separately on both morning and afternoon items, in the total sample and also within age subgroups, comparing results between age groups to inform any differences in item-scale groupings in different age populations	Eigenvalues (and accompanying scree plots) were produced to assess suitable number of dimensions and factor loadings (> 0.40) for each item were examined Modification indices (MIs) were also assessed, to assess the extent to which items and/or domains were co-dependent and thus potentially redundant. Items and/or domains with MIs greater than or equal to fifteen were considered further [15] Model fit was assessed using Bentler’s CFI, RMSEA and SRMR. According to the a priori specification, model fit was considered ‘good’ if CFI > 0.95, RMSEA < 0.08 and SRMR < 0.08 [16]
Item response theory (IRT)	IRT was conducted to provide insight into the appropriateness of the response scale and the adequacy of item discrimination using the Graded Response Model (GRM) Item information curves (IICs) were examined graphically to evaluate how reliably the individual items and the measure as a whole estimated the construct over the entire scale range	Using the GRM, S-X^2^ fit statistic was examined to assess the differences between observed and expected response proportions for each test score values with statistically significant values (p < 0.01) indicating items with potential misfit. Chi-square statistics to evaluate local dependence (values of > 10 indicating likely local dependence)
Internal consistency reliability	Internal consistency reliability, concerned with the homogeneity of items belonging to the same domain was evaluated in all age subgroups as well as in the total sample The alpha-if-item-deleted method was also used to assess whether the internal consistency of the summary score would improve with the removal of each item in turn	Internal consistency reliability was evaluated using Cronbach’s alpha coefficient (> 0.70 for good internal consistency) [17]
Test-retest reliability (TRT)	TRT was evaluated by examining the stability of scores between two daily consecutive assessments. Given the acute nature of the common cold, participants were defined as stable if they had ‘no change’ in their overall cold severity based on their CGI-S scores between Visit 1 and Day 1 and between Visit 1 and Day 2	Intraclass correlation coefficients (ICCs) were calculated and an ICC of 0.70 or greater for the stable group was considered evidence of good TRT [18]
Known groups validity	Known groups validity was assessed by comparing differences in the chest-related ePRO diary scores among participants who differed on health/disease related variables based on the CGI-S and CCSQ measures A mixed model repeated measures analysis was used to examine the relationship between the CGI-S and the total chest-related ePRO diary summary scores using all ten days of diary data available, with the CGI-S treated as a continuous predictive variable and the chest ePRO as the outcome variable. A sensitivity analysis was also performed in which the CGI-S was treated as a categorical variable. In addition to the above analyses where known groups were defined using the CGI-S, groups were additionally defined using the CCSQ summary score made up of the mean of all CCSQ items. For each day that the CCSQ was collected (Days 2, 5 and 8), a mixed model repeated measures analysis was used to examine the relationship between the CCSQ and the total chest-related ePRO diary scores. The CCSQ was used as the predictive variable and treated as a continuous variable with the chest-related ePRO diary score as the outcome variable	T-tests were used for comparisons of pairs of groups to evaluate statistically significant differences (p < 0.05) in chest-related ePRO diary scores between the subgroups
Concurrent validity	Concurrent validity was evaluated by examining the correlation between the scores of the chest-related ePRO diary and the CCSQ on Days 2, 5 and 8	Domains assessing similar or related concepts were expected to correlate at 0.40 or higher
Ability to detect change	The analysis described and compared changes in the chest-related ePRO diary scored between participants considered ‘improved’, ‘no change’ or ‘worsened’ as assessed by ratings on the CGI-C and CGI-S scores, to demonstrate that any observed changes in the chest-related ePRO diary scores corresponded with changes in external criteria	The mean changes in the symptom scores were computed between the change groups using paired t-tests as the ‘no change’ and ‘worsened’ groups were collapsed due to a low sample size Effect sizes (ES), standardised response mean (SRM) and Guyatt’s statistic were calculated to evaluate the magnitude of changes over time in each group. ES were interpreted in line with Cohen’s guidance (0.20: small changes, 0.50: moderate changes, and 0.80 large changes) [19]

### Exit interviews

Thirty (6–8 years [n = 6], 9–11 years [n = 8], 12–17 years [n = 7] and 18+ years [n = 9]) participants also took part in exit interviews at the end of Study Visit 2. Interviews included a brief CE section to elicit information on language used by patients to describe cold symptoms, followed by a CD section [11] to debrief the ePRO diary and identify any challenges relating to device usability and feasibility of completing the measure twice daily for 10 days. All exit interviews were audio recorded and transcribed verbatim. Transcripts were then qualitatively analyzed using thematic analysis methods and Atlas.ti software [12].

## Results

### Sample demographic and clinical characteristics

Of 200 screened participants, 195 participants across age groups (6–8 years [n = 42], 9–11 years [n = 47], 12–17 years [n = 55] and 18 + [n = 51) met the inclusion and exclusion criteria and completed at least five days of ePRO data. There were more female participants (64.6%) than male (35.4%), and the majority were non-Hispanic/Latino ethnicity (83.1%). The most represented races were ‘white’ (40.5%) and ‘black/African American’ (40.5%). Thus, there was good demographic diversity in the sample.

In physical assessments, areas most frequently classified as ‘abnormal’ were the nose (n = 141, 72.3%) and throat (n = 93, 47.7%). The most reported additional health conditions in the sample were allergies (n = 31, 12.4%) and head/ear/eye/nose/throat conditions (n = 31, 12.4%). Thirty-five (13.9%) participants reported taking medications for their cough/cold and fifteen (6.0%) for aches/ pains.

For baseline CGI-S scores, 95 participants (48.7%) rated their cold as ‘very bad’ and 100 (51.3%) as ‘bad’. Thus, at baseline most of the sample were reporting burdensome cold symptoms, in line with the inclusion criteria.

### Quality of completion

Quality of completion was very good overall (≤ 10.9% missing completions across all visits). Most participants did not miss either a single morning completion (n = 147, 76.2%) or afternoon completion (n = 117, 60.6%). Out of those who missed a morning or afternoon completion, most only missed one. No participants missed more than four days. Afternoon completions were slightly more frequently missed in general.

### Item response distributions

Tables and stacked bar charts summarizing the item response distributions for the morning and afternoon diary for each of the different age groups for Days 1, 5 and 10 are provided in Additional file 1: Supplementary Files. Across all age groups, the results indicated a good spread of responses across the response scale for all ten items across the days. There was no evidence of more favourable distributions of responses for any particular items. Comparison of Days 1 to 10 across all age groups demonstrated responses gradually shifting down the scale, indicating symptom improvement, as would be expected given the typical trajectory of common cold chest symptoms. Floor effects were present in all items at Day 10, again consistent with expectations. No floor or ceiling effects were present at baseline or Days 1–5, where their presence would be a cause for concern.

### Mean chest-related ePRO diary item scores

**Fig. 3:**
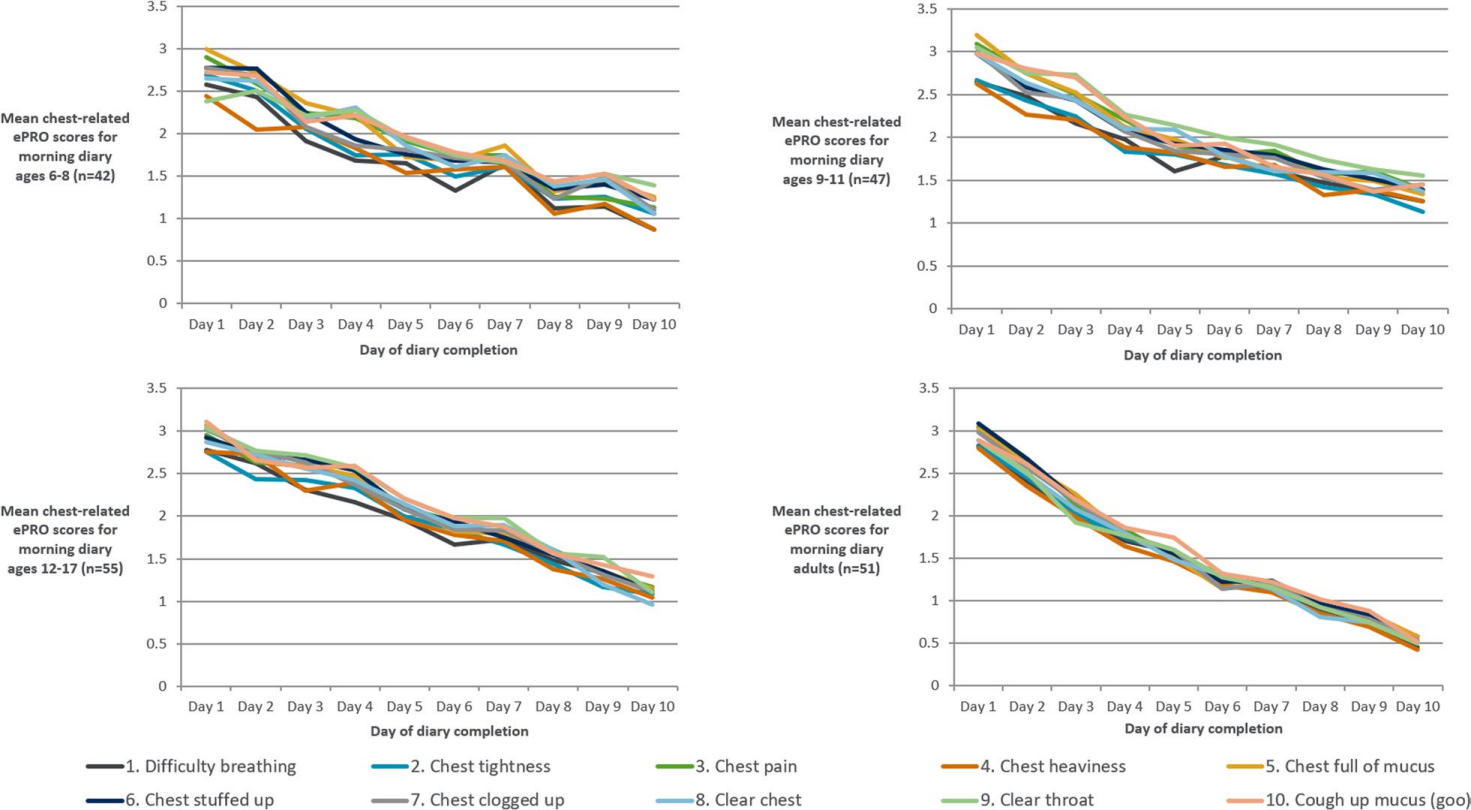
Mean chest-related ePRO scores for the morning diary across all age groups from Day 1 to Day 10

All mean item scores for all age groups demonstrated a gradual improvement between Day 1 and Day 10, indicating symptom improvement, and providing evidence supporting sensitivity of the items to changes in the symptoms of chest congestion over time. Although the trajectory of chest symptoms over time was broadly comparable in children, adolescents and adults, based on descriptive analyses, the adult group mean scores showed a slightly steeper rate of improvement suggesting potentially faster recovery in adults compared to children or adolescents (Fig. 3). There was also some evidence of mean morning diary item scores being consistently slightly higher than afternoon scores, particularly in the 6–11 age groups. However, by Day 10 any differences between morning and afternoon scores were negligible. Mean chest-related ePRO diary afternoon scores are provided in Additional file 1: Supplementary Files.

### Item performance

#### Inter-item correlations

The majority of inter-item correlations were moderate to high indicating that most items are assessing related, but not redundant concepts. Inter-item correlations between five item pairs (Table 3) were very high (Pearson correlation > 0.80), and the items were flagged for further consideration. The correlations between items 6 (‘chest stuffed up’) and 7 (‘chest clogged up’) were generally above 0.80 for all age groups for both morning and afternoon assessments. Similarly, item 1 (‘difficulty breathing’) was highly correlated (r = 0.844) with item 2 (‘chest tightness’). This trend was observed in all age groups but was particularly prominent in afternoon assessments and suggests that the items are closely related rather than suggesting redundancy. Afternoon item 4 (‘chest heaviness’) also highly correlated with afternoon items 1 (0.628–0.861) and 2 (0.733–0.861) across age groups. Low (< 0.40) correlations were observed between morning items 3 (‘how much has your chest hurt when you’ve coughed?’) and 10 (‘how hard was it to cough up mucus (goo) from your chest?’) but this was expected as the concepts assessed by the items are relatively unrelated. Inter-item correlations were slightly higher in the adolescent age group (12–17) compared to the other age groups. Full correlation matrices for morning and afternoon items are provided in Additional file 1: Supplementary Files.

**Table 3:** Summary of inter-item correlations > 0.80

Item	Correlation	Item
Morning item 6: chest stuffed up	0.845	Morning item 7: chest clogged up
Afternoon item 1: difficulty breathing	0.844	Afternoon item 2: chest tightness
Afternoon item 2: chest tightness	0.807	Afternoon item 4: chest heaviness
Afternoon item 5: chest feels full	0.812	Afternoon item 7: chest clogged up
Afternoon item 6: chest stuffed up	0.823	Afternoon item 7: chest clogged up

##### Differential item functioning

For most items, there was no evidence of DIF, and in those small number of instances where DIF was present, it was uniform in nature. Uniform DIF was only present for item 9 (‘clear throat’) in the morning diary when comparing the 9–11 year-olds (p < 0.001) and adults, and when comparing the 12–17-year-olds and the adults (p = 0.006). Results from these analyses indicated that participants across the different age groups responded to the chest-related ePRO diary items in a functionally equivalent way, suggesting that scores obtained on the chest-related ePRO diary are directly comparable between the different age groups.

### Exploratory factor analysis (EFA)

Exploratory factor analysis (EFA) was performed separately on morning and afternoon items. For morning items, all eigenvalues except the first fell below one, and scree plots levelled off between factors 1 and 2 in the total sample as well as in both age subgroups (6–11 and 12 +) supporting a one-factor solution. All items also loaded highly onto a single factor with similar magnitudes of factor loading. In all age groups, the model met two of the three fit criteria, with the RMSEA slightly exceeding the prespecified 0.10 threshold. High MIs were observed between items 6 (‘chest stuffed up’) and 7 (‘chest clogged up’) in both the total sample (MI 36.97) and the 12 + age group (MI 20.90), suggesting potential redundancy.

For afternoon items, all eigenvalues except the first similarly fell below one, and scree plots levelled off between factors 1 and 2 in the total sample and in both age subgroups (6–11 and 12 +), supporting a one factor solutions. Similar to morning item EFA, all items loaded highly onto a single factor with similar factor loading magnitudes. The model met two out of three fit criteria in the total sample and both age subgroups, with RMSEA exceeding the prespecified 0.10 threshold for good fit. One high MI (28.90) was observed between item 1 ‘difficulty breathing’ and item 2 ‘chest tightness’ in the total sample. Factor loadings and model fit indices for morning and afternoon EFA are provided in Table 4. All eigenvalues, scree plots and high item MIs for the total sample for morning and afternoon item EFA are provided in Additional file 1: Supplementary Files.

**Table 4:** Exploratory factor analysis of chest-related ePRO morning and afternoon diary items

	Factor loadings		
Items	Total sample (N = 195)	6–11 year olds (n = 89)	12+ year olds (N = 106)
*Morning items*			
Morning 1 (difficulty breathing)	0.888	0.858	0.858
Morning 2 (chest tightness)	0.886	0.858	0.858
Morning 3 (chest pain)	0.778	0.718	0.718
Morning 4 (chest heaviness)	0.878	0.879	0.879
Morning 5 (chest full of mucus)	0.865	0.809	0.809
Morning 6 (chest stuffed up)	0.910	0.889	0.889
Morning 7 (chest clogged up)	0.942	0.903	0.903
Morning 8 (clear chest)	0.891	0.891	0.891
Morning 9 (clear throat)	0.766	0.789	0.789
Morning 10 (cough up mucus/goo)	0.802	0.779	0.779
*Model fit indices*			
CFI	0.990	0.990	0.994
RMSEA	0.113	0.095	0.112
SRMR	0.035	0.044	0.039
*Afternoon items*			
Afternoon 1 (difficulty breathing)	0.904	0.907	0.913
Afternoon 2 (chest tightness)	0.929	0.940	0.923
Afternoon 3 (chest pain)	0.832	0.811	0.863
Afternoon 4 (chest heaviness)	0.892	0.871	0.921
Afternoon 5 (chest full of mucus)	0.886	0.836	0.940
Afternoon 6 (chest stuffed up)	0.924	0.907	0.970
Afternoon 7 (chest clogged up)	0.938	0.919	0.952
Afternoon 8 (clear chest)	0.874	0.823	0.926
Afternoon 9 (clear throat)	0.786	0.833	0.744
Afternoon 10 (cough up mucus/goo)	0.836	0.788	0.872
*Model fit indices*			
CFI	0.987	0.980	0.995
RMSEA	0.135	0.147	0.114
SRMR	0.035	0.051	0.041

### Item response theory (IRT)

S-X^2^ statistics for morning items indicated potential misfit of one item in the total sample (item 10 ‘cough up mucus/goo’, p = 0.006). No significant p-values (< 0.01) were observed for any of the items within the age subgroups. There was no evidence of local dependence between items across each of the different age groups. For afternoon items, misfit was indicated for item 1 (‘difficulty breathing’) in the 6–11 age group (p = 0.0015) and 12 + age group (p = 0.0007), as well as item 10 (‘cough up mucus/goo’) in the 6–11 year old age subgroup (p = 0.0039). Tables detailing S-X^2^ statistics of all morning and afternoon items are included in Additional file 1: Supplementary Files.

**Fig. 4:**
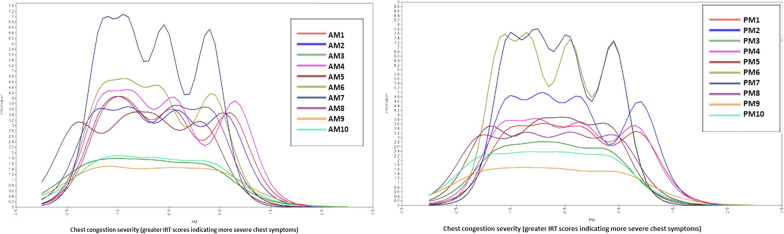
IICs for AM and PM items for the total study sample

When evaluating IICs for morning items, items 4 (‘chest heaviness’), 6 (‘chest stuffed up’) and 7 (‘chest clogged up’) provided the most information in the total sample. The lowest amount of information was provided by items 3 (‘chest pain’) 9 (‘clear throat’) and 10 (‘cough up mucus/goo’). In evaluations of afternoon items, results were similar with items 6 (‘chest stuffed up’) and 7 (‘chest clogged up’) providing the greatest amount of information, and items 3 (‘chest pain’), 9 (‘clear throat’) and 10 (‘cough up mucus/goo’) providing the least amount of information. IICs for morning and afternoon items in the total sample are provided below in Fig. 4. IICs for each of the age subgroups are included in Additional file 1: Supplementary Files.

### Item reduction to finalise chest-related ePRO diary

Item deletion decisions were driven by the performance of items in the item-level analyses, dimensionality analyses, the previous qualitative findings and clinical judgement. Coming out of this process, three items were deleted based on poor item performance, poor face validity/clinical relevance and/or conceptual redundancy: items 3 (‘chest pain’), 6 (‘chest stuffed up’) and 9 (‘clear throat’), resulting in the 7-item chest-related ePRO diary. All analyses described hereon were conducted on the 7-item version of the chest-related ePRO diary. Further details on item-deletion decisions are provided in Additional file 1: Supplementary Files.

### Scoring algorithm development

**Fig. 5:**
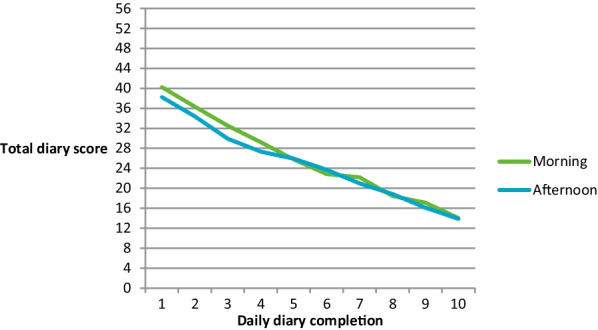
Total score of morning vs. afternoon chest-related ePRO diary summary scores from Day 1 to Day 10 (N = 195)

Figure 5 presents the trajectory of the total morning and afternoon diary item scores over the ten day at-home completion period. It is evident that there was very little discrepancy between participants’ morning and afternoon item scores. These additional results suggest that it would be appropriate to either score the morning and afternoon scores together or create separate scores. Based on these findings, it was decided that a total daily summary score would be created that combined the morning and afternoon items (n = 14) into a single score based on the summed total of all item responses (0–4). This creates a total score that can range between 0 and 56, with higher scores indicating more severe chest-related symptoms throughout the day.

All subsequent analyses (reliability, known-groups analyses and ability to detect change) were performed on this sum score of all 14 remaining morning and afternoon items.

### Reliability and validity

#### Internal consistency reliability

Internal consistency of the chest-related ePRO was very high when examined in the total sample and within age sub-groups (Cronbach’s alpha: 0.957–0.975). Although the alpha coefficients were so high as to suggest some potential redundancy among the items, calculation of the alpha coefficient with each item deleted in turn resulted in lower Cronbach’s alpha values suggesting that each item improved the reliability of the summary score supporting retention of all items. Details of internal consistency reliability analysis are provided in Additional file 1: Supplementary Files.

#### Test-retest reliability

Test-retest reliability of the ePRO summary score was assessed by defining stable participants as those with 1) “no change” between Visit 1 and Day 1 and 2) those with “no change” between Visit 1 and Day 2 on the CGI-S (Table 5). Overall, the summary score demonstrated strong test-retest reliability in the total sample and across all age groups. ICCs were slightly higher for the shorter time period, as expected with Visit 1 to Day 1 results ranging from 0.843–0.-893 and Visit 1 to Day 2 results ranging from 0.783–0.793. Thus, all results exceeded the threshold of 0.70 providing evidence of strong test-retest reliability.

**Table 5:** Test-retest reliability for the chest-related ePRO summary score using the definitions based on the CGI-S

	Age group	N	Reliability (ICC) [1]	95% confidence intervals	
				Lower	Upper
*Stable sample defined based on “no change” between Visit 1 and Day 1 on the CGI-S*					
ePRO summary score	Total sample	84	0.869	0.805	0.913
	6–11	40	0.843	0.723	0.913
	12+	44	0.893	0.812	0.940
*Stable sample defined based on “no change” between Visit 1 and Day 2 on the CGI-S*					
ePRO summary score	Total sample	70	0.786	0.677	0.861
	6–11	30	0.783	0.595	0.890
	12+	40	0.793	0.642	0.884

### Construct validity

#### Known groups validity

**Fig. 6:**
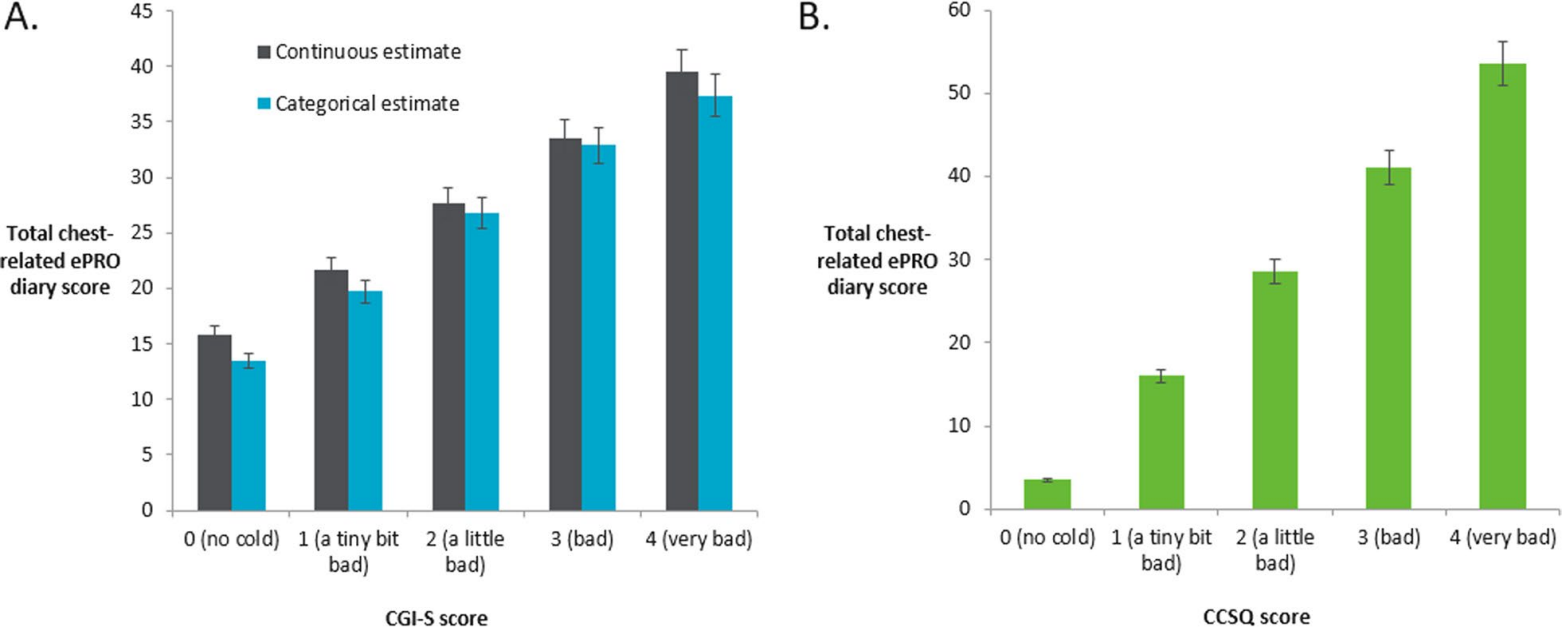
Known groups validity of the chest-related ePRO as defined based on CGI-S (**A**) and CCSQ (**B**) responses

Chest-related ePRO scores were compared between groups according to their overall cold severity (as defined based on responses on the CGI-S and CCSQ). Both analyses demonstrated strong known-groups validity of the chest-related ePRO. There were statistically significant differences among the five CGI-S and CCSQ response groups (p < 0.05) with the chest-related ePRO summary total score estimates monotonically increasing across the severity groups in a logical pattern as expected (with no overlap in confidence intervals) with both the CGI-S (Fig. 6A) and CCSQ (Fig. 6B). This was irrespective of whether the CGI-S was treated as continuous or categorical.

#### Concurrent validity

Correlations between the chest-related ePRO diary summary score with the CCSQ items were moderate to high (> 0.50) for all items and at all time points (Table 6). While all the correlation coefficients were moderate to high, as expected, there were slightly higher correlations between the chest-related ePRO diary summary score and the CCSQ items assessing chest congestion (range 0.838–0.920), compared to the correlations with the CCSQ items evaluating less closely related constructs, such as nasal congestion (range 0.628–0.789). Thus, these high correlations and the logical pattern of correlations overall support the concurrent validity of the chest-related ePRO diary. Findings were also similar when examined within age subgroups.

**Table 6:** Concurrent validity: polyserial Correlations between the chest-related ePRO total summary score and the CCSQ for all participants (N = 195)

CCSQ ITEM		Polyserial Correlations with ePRO Total Score		
		Day 2 ePRO Total Score (n = 190)	Day 5 ePRO Total Score (n = 187)	Day 8 ePRO Total Score (n = 184)
Cough	AM1 (cough frequency)	0.716	0.804	0.789
	AM4 (cough severity)	0.699	0.768	0.795
	AM5 (cough frequency)	0.707	0.759	0.807
	PM1 (cough severity)	0.768	0.760	0.791
	PM12 (cough frequency)	0.675	0.744	0.798
Nasal congestion	AM2 (stuffy nose)	0.641	0.772	0.803
	AM3 (runny nose)	0.608	0.757	0.801
	AM9 (stuffy nose)	0.638	0.820	0.810
	AM10 (clear nose)	0.588	0.683	0.756
	PM2 (runny nose)	0.650	0.686	0.763
	PM3 (stuffy nose)	0.645	0.758	0.833
	PM4 (clear nose)	0.640	0.690	0.767
	PM10 (blow nose)	0.654	0.758	0.737
	M11 (stuffy nose)	0.617	0.756	0.807
Chest congestion	AM6 (breathe air deep chest)	0.844	0.908	0.920
	AM11 (chest tightness)	0.851	0.902	0.868
	PM5 (chest tightness)	0.838	0.904	0.906
	PM13 (breathe air deep chest)	0.855	0.887	0.907
Head, face, and body pains	AM12 (pain face nose)	0.688	0.752	0.745
	AM14 (head hurt)	0.681	0.727	0.736
	AM8 (headache)	0.669	0.762	0.741
	AM15 (arm/leg ache)	0.628	0.723	0.717
	PM6 (pain face, eyes/nose)	0.699	0.763	0.732
	PM8 (head hurt)	0.670	0.693	0.727
	PM9 (arm/leg ache)	0.642	0.682	0.725
	PM14 (tight eyes/nose)	0.756	0.789	0.762
	PM16 (headache)	0.677	0.710	0.724
	PM17 (arm/leg ache)	0.654	0.670	0.724
Sore throat	AM7 (pain swallow)	0.706	0.804	0.821
	AM13 (sore throat)	0.624	0.799	0.765
	PM7 (sore throat)	0.633	0.813	0.785
	PM15 (pain swallow)	0.709	0.831	0.760

*Where the number of participants is not equal to the total sample, this was due to missing completions

### Ability to detect change

Changes in the chest-related ePRO summary score over time were compared among participants defined as ‘improved’ or ‘stable/worsened’ as assessed on the CGI-S (between Visit 1 to day 4 and Day 10) and CGI-C (at days 2, 5 and 8 compared to Visit 1). ‘Stable’ and ‘worsened’ groups were collapsed due to the very small number of participants defined as ‘worsened’. Overall, the results showed that the chest-related ePRO is able to detect changes in symptoms over the course of a common cold irrespective of the rating used to define change, with statistically significant differences between the ‘improved’ and ‘stable/worsened’ groups (Table 7). Although the SES were in some cases ‘large’ even in the ‘stable/worsened’ group (range: 0.03 to -1.18), they were consistently much larger in the ‘improved’ group (range: -1.13 to -3.26).

**Table 7:** Ability to detect change: mean change scores and Standardized Effect Sizes (SES) on the chest-related ePRO summary score with change groups based on change in the CGI-S between Visit 1 (baseline) to Day 5 (n = 187) and Day 10 (n = 177) and CGI-C at Day 2 (n = 191), Day 5 (n = 187) and Day 8 (n = 184)

Group	N	Mean change (SD)	Within-group p-value	Between group p-value	SES
*Days 1–5 (stable participants defined as those with no change on the CGI-S)*					
Improved	126	− 20.2 (13.7)	< 0.001	< 0.001	− 1.82
Stable/worsened	46	− 3.5 (12.5)	0.062		− 0.33
*Days 1–10 (stable participants defined as those with no change on the CGI-S)*					
Improved	136	− 31.4 (15.0)	< 0.001	< 0.001	− 2.79
Stable/worsened	18	0.4 (10.2)	0.873		0.03
*Days 1–5 (stable participants defined as those with no change or 1 point change on the CGI-S)*					
Improved	73	− 25.5 (13.6)	< 0.001	< 0.001	− 2.24
Stable/worsened	99	− 8.6 (12.3)	< 0.001		− 0.78
*Days 1–10 (stable participants defined as those with no change or 1 point change on the CGI-S)*					
Improved	110	− 34.8 (13.4)	< 0.001	< 0.001	− 3.14
Stable/worsened	44	− 10.0 (14.7)	< 0.001		− 0.76
*Day 2 (stable participants defined as those who reported being ‘Unchanged’ on the CGI-C)*					
Improved	72	− 12.5 (11.5)	< 0.001	< 0.001	− 1.13
Stable/worsened	99	− 2.1 (9.8)	0.039		− 0.19
*Day 5 (stable participants defined as those who reported being ‘Unchanged’ on the CGI-C)*					
Improved	109	− 20.1 (15.0)	< 0.001	< 0.001	− 1.81
Stable/worsened	62	− 8.2 (13.0)	< 0.001		− 0.90
*Day 8 (stable participants defined as those who reported being ‘Unchanged’ on the CGI-C)*					
Improved	119	− 28.9 (14.4)	< 0.001	< 0.001	− 2.61
Stable/worsened	47	− 8.2 (13.8)	< 0.001		− 0.67
*Day 2 (stable participants defined as those who reported being ‘Unchanged’ or ‘A little better/worse’ on the CGI-C)*					
Improved	10	− 23.7 (15.1)	< 0.001	< 0.001	− 2.06
Stable/worsened	161	− 5.4 (10.6)	< 0.001		− 0.48
*Day 5 (stable participants defined as those who reported being ‘Unchanged’ or ‘A little better/worse’ on the CGI-C)*					
Improved	42	− 27.9 (15.7)	< 0.001	< 0.001	− 2.28
Stable/worsened	129	− 11.8 (13.0)	< 0.001		− 1.13
*Day 8 (stable participants defined as those who reported being ‘Unchanged’ or ‘A little better/worse’ on the CGI-C)*					
Improved	71	− 34.9 (12.3)	< 0.001	< 0.001	− 3.26
Stable/worsened	95	− 14.1 (14.5)	< 0.001		− 1.18

### Exit interviews

Most participants provided positive feedback on the chest-related ePRO, with eighteen (n = 18/24, 75.0%) participants mentioning at least one feature they liked, and all those asked (n = 21/21 100.0%) finding the alarm function on the devices helpful.

Overall, participants reported finding it easy to remember and rate their symptoms during the study. Nine participants out of 25 asked (36%) (aged 6–8 years [n = 3], 9–11 years [n = 3], 12–17 years [n = 2], 18 + years [n = 1]) reported that they may find it challenging to recall how their cold had felt over the specified recall period.

Exit interview results also provided further evidence of good understanding for majority of the items, consistent with previous qualitative research. Item understanding increased with age, with total understanding within age groups increasing from 60% in the 6–8-year-old group to 78% in 12–17-year-olds; however, such age differences should be interpreted with caution, given the relatively small qualitative sample size. A detailed breakdown of item understanding by participant age group is provided in Additional file 1: Supplementary Files.

## Discussion

The findings of this psychometric evaluation study provide evidence that the chest-related ePRO diary is valid, reliable and is able to detect change over time as an assessment of chest-related symptoms of a URTI such as the common cold in pediatric and adolescent populations, and that only minor differences exist in the URTI trajectories between different age groups. Missing data levels across the ten day completion period were very low, providing evidence that the diary was not burdensome to complete twice daily and the ePRO format is a feasible tool to encourage a good level of completion.

Item response distributions provided evidence that the items can capture the range of chest congestion severities and are able to reflect changes in symptoms of chest congestion over time. Floor effects were observed at Day 10, but this is consistent with the duration of URTI symptoms in published research, and thus provides support for the validity of the measure [13]. The decrease in symptom severity observed on the chest-related ePRO across the study period was also in line symptom trajectories illustrated in past research on The Canadian Acute Respiratory Illness and Flu Scale (CARIFS) [14] and CCSQ [5]. However, as neither the CARIFS nor CCSQ measure chest congestion specifically, the chest-related ePRO could be used to address this gap in conceptual coverage in instances where assessment of chest congestion symptoms is important.

The EFA conducted as part of this study also provides strong evidence of the item-scale structure of the chest-related ePRO diary (i.e., a single, unidimensional score). The measure also demonstrated strong concurrent validity with higher correlations with related items on the CCSQ, than with less closely related items. Test-retest reliability analyses were conducted in line with FDA recommendations with strong results indicating good test-retest reliability [4]. Due to age and developmental differences, not least in the ability to understand PRO items, any PRO used in a pediatric population must demonstrate strong psychometric evidence within narrow age bands [3, 4, 7]. Such data have been generated as part of this study, providing reassurance that the measure performs equally strongly in both the 6–11 and 12–17 age groups.

Although the evidence generated as part of this study for the chest-related ePRO diary provides robust initial evidence of its psychometric properties, some limitations must be acknowledged. Although item performance was strong overall, known groups analyses were performed in groups defined based on the CGI-S, which assesses overall cold symptoms rather than chest-specific symptoms, and thus the varying resolution times of different symptoms of a URTI may complicate the analysis.

While the study benefited from a racially diverse sample, all participants were resident in the USA. Further studies in other countries/cultures following translation and linguistic validation is recommended in the future if the measure is to be used in international clinical trials or research studies, as differences between education systems across countries may contribute to children’s reading level irrespective of age and alter item understanding and interpretation [7]. It is arguably a strength of the study that the study criteria did not require participants to adhere to or refrain from any specific treatment for their URTI, and that analyses allowed for comparisons between pediatric, adolescent, and adult populations. Further, the psychometric evidence presented here is corroborated by thorough qualitative evidence supporting the instrument’s content validity [6]. Nevertheless, further inclusion of and assessment of the performance of the chest-related ePRO in interventional studies would be beneficial to confirm that the measurement properties are robust in that specific context of use, and specifically to allow estimation of meaningful change thresholds to aid interpretation of changes in score over time in response to treatment. Finally, participants also completed the measure on a handheld, ePRO device—if migrated to pen/paper or another electronic mode of administration, it would be recommended to confirm the psychometric properties are equally strong. The chest-related ePRO diary measure is available for educational, research and clinical use at no cost.

## Conclusion

The findings of this study demonstrate that the chest-related ePRO diary provides a valid and reliable assessment of chest congestion symptoms in children and adolescents that has the ability to detect changes over time. The study also provides evidence that patients of different ages can adhere to 10-day completion using a handheld ePRO device, supporting the use of the chest-related ePRO diary in future clinical trials.

## Supplementary Information

**Additional file 1**. Supplementary files.

## Data Availability

The data described in this article are not publicly available in further detail beyond that provided in the manuscript and the extensive Additional file 1: Supplementary Files.
