# Supplementary material for: Psychometric validation and testing of the 10-item pediatric daily chest-related electronic patient reported outcome (ePRO) diary
Source: J Patient Rep Outcomes. 2023 Jan 25;7:6. doi: 10.1186/s41687-023-00546-2 (PMC9877252; doi:10.1186/s41687-023-00546-2)
Supplement: Supplementary file 1 — Additional file 1. Supplementary files. [file 41687_2023_546_MOESM1_ESM.docx]

**Psychometric Validation and Testing of the 10-Item Pediatric Daily Chest-Related Electronic Patient Reported Outcome (ePRO) Diary – Supplementary Files**

## Item response distributions

Supplementary figure 1. Item response distribution for the morning items of the chest-related ePRO diary scores at Day 1 in the total sample (n=195)

Supplementary figure 2. Item response distributions for the morning items of the chest-related ePRO diary scores at Day 5 in the total sample (n=195)

Supplementary figure 3. Item response distributions for the morning items of the chest-related ePRO diary scores at Day 10 in the total sample (n=195)

| Supplementary table 1. Item distribution: chest-related ePRO morning/afternoon diary for all participants at Day 1 (n=191) | | | | | | | | | | |
| --- | --- | --- | --- | --- | --- | --- | --- | --- | --- | --- |
| ePRO daily diary | Item response category [1] | | | | | | | | | |
|  | Morning diary | | | | | Afternoon diary | | | | |
|  | 0 | 1 | 2 | 3 | 4 | 0 | 1 | 2 | 3 | 4 |
| 1. How hard was it to breathe air deep into your chest? | 9  (4.9%) | 13  (7.0%) | 47 (25.4%) | 68 (36.8%) | 48 (25.9%) | 14  (7.8%) | 23 (12.8%) | 45 (25.0%) | 60 (33.3%) | 38 (21.1%) |
| 2. How tight did your chest feel? | 5 (2.7%) | 18  (9.7%) | 51 (27.6%) | 57 (30.8%) | 54 (29.2%) | 12 (6.7%) | 21 (11.7%) | 45 (25.0%) | 66 (36.7%) | 36 (20.0%) |
| 3. How much has your chest hurt when you’ve coughed? | 7 (3.8%) | 13  (7.0%) | 31 (16.8%) | 56 (30.3%) | 78 (42.2%) | 11 (6.1%) | 18 (10.0%) | 27 (15.0%) | 55 (30.6%) | 69 (38.3%) |
| 4. How heavy did your chest feel? | 7 (3.8%) | 18  (9.7%) | 47 (25.4%) | 70 (37.8%) | 43 (23.2%) | 11 (6.1%) | 20 (11.1%) | 49 (27.2%) | 60 (33.3%) | 40 (22.2%) |
| 5. How much did your chest feel full of mucus (goo)? | 2 (1.1%) | 17 (9.2%) | 26 (14.1%) | 61 (33.0%) | 79 (42.7%) | 7  (3.9%) | 22 (12.2%) | 32 (17.8%) | 53 (29.4%) | 66 (36.7%) |
| 6. How stuffed up did your chest feel? | 6 (3.2%) | 13 (7.0%) | 32 (17.3%) | 68 (36.8%) | 66 (35.7%) | 9  (5.0%) | 13 (7.2%) | 41 (22.8%) | 59 (32.8%) | 58 (32.2%) |
| 7. How clogged up did your chest feel? | 5 (2.7%) | 13 (7.0%) | 37 (20.0%) | 61 (33.0%) | 69 (37.3%) | 11 (6.1%) | 15 (8.3%) | 34 (18.9%) | 62 (34.4%) | 58 (32.2%) |
| 8. How hard was it to clear your chest? | 8 (4.3%) | 13 (7.0%) | 47 (25.4%) | 47 (25.4%) | 70 (37.8%) | 5  (2.8%) | 17 (9.4%) | 43 (23.9%) | 56 (31.1%) | 59 (32.8%) |
| 9. How hard was it to clear your throat? | 10 (5.4%) | 9 (4.9%) | 45 (24.3%) | 54 (29.2%) | 67 (36.2%) | 9  (5.0%) | 15 (8.3%) | 33 (18.3%) | 63 (35.0%) | 60 (33.3%) |
| 10. How hard was it to cough up mucus (goo) from your chest? | 10 (5.4%) | 9 (4.9%) | 36 (19.5%) | 57 (30.8%) | 73 (39.5%) | 7  (3.9%) | 17 (9.4%) | 35 (19.4%) | 53 (29.4%) | 68 (37.8%) |
| *[1] Missing data excluded from this analysis so that the percentages are calculated based on those patients who completed the item.* | | | | | | | | | | |

| Supplementary table 2. Item distribution: chest-related ePRO morning/afternoon diary for all participants at Day 5 (n=187) | | | | | | | | | | |
| --- | --- | --- | --- | --- | --- | --- | --- | --- | --- | --- |
| ePRO daily diary | Item response category [1] | | | | | | | | | |
|  | Morning diary | | | | | Afternoon diary | | | | |
|  | 0 | 1 | 2 | 3 | 4 | 0 | 1 | 2 | 3 | 4 |
| 1. How hard was it to breathe air deep into your chest? | 40 (21.9%) | 40 (21.9%) | 57 (31.1%) | 25 (13.7%) | 21 (11.5%) | 43 (24.3%) | 39 (22.0%) | 52 (29.4%) | 22 (12.4%) | 21 (11.9%) |
| 2. How tight did your chest feel? | 38 (20.8%) | 42 (23.0%) | 48 (26.2%) | 32 (17.5%) | 23 (12.6%) | 34 (19.2%) | 43 (24.3%) | 51 (28.8%) | 28 (15.8%) | 21 (11.9%) |
| 3. How much has your chest hurt when you’ve coughed? | 34 (18.6%) | 38 (20.8%) | 56 (30.6%) | 30 (16.4%) | 25 (13.7%) | 39 (22.0%) | 45 (25.4%) | 35 (19.8%) | 29 (16.4%) | 29 (16.4%) |
| 4. How heavy did your chest feel? | 39 (21.3%) | 45 (24.6%) | 52 (28.4%) | 25 (13.7%) | 22 (12.0%) | 40 (22.6%) | 43 (24.3%) | 50 (28.2%) | 22 (12.4%) | 22 (12.4%) |
| 5. How much did your chest feel full of mucus (goo)? | 33 (18.0%) | 47 (25.7%) | 43 (23.5%) | 36 (19.7%) | 24 (13.1%) | 32 (18.1%) | 46 (26.0%) | 44 (24.9%) | 26 (14.7%) | 29 (16.4%) |
| 6. How stuffed up did your chest feel? | 33 (18.0%) | 47 (25.7%) | 46 (25.1%) | 31 (16.9%) | 26 (14.2%) | 29 (16.4%) | 44 (24.9%) | 51 (28.8%) | 23 (13.0%) | 30 (16.9%) |
| 7. How clogged up did your chest feel? | 36 (19.7%) | 44 (24.0%) | 44 (24.0%) | 32 (17.5%) | 27 (14.8%) | 33 (18.6%) | 37 (20.9%) | 47 (26.6%) | 29 (16.4%) | 31 (17.5%) |
| 8. How hard was it to clear your chest? | 35 (19.1%) | 32 (17.5%) | 60 (32.8%) | 29 (15.8%) | 27 (14.8%) | 34 (19.2%) | 34 (19.2%) | 52 (29.4%) | 23 (13.0%) | 34 (19.2%) |
| 9. How hard was it to clear your throat? | 34 (18.6%) | 33 (18.0%) | 51 (27.9%) | 34 (18.6%) | 31 (16.9%) | 32 (18.1%) | 37 (20.9%) | 45 (25.4%) | 31 (17.5%) | 32 (18.1%) |
| 10. How hard was it to cough up mucus (goo) from your chest? | 34 (18.6%) | 40 (21.9%) | 40 (21.9%) | 37 (20.2%) | 32 (17.5%) | 31 (17.5%) | 31 (17.5%) | 52 (29.4%) | 28 (15.8%) | 35 (19.8%) |
| *[1] Missing data excluded from this analysis so that the percentages are calculated based on those patients who completed the item.* | | | | | | | | | | |

| Supplementary table 3. Item distribution: chest-related ePRO morning/afternoon diary for all participants at Day 10 (n=177) | | | | | | | | | | |
| --- | --- | --- | --- | --- | --- | --- | --- | --- | --- | --- |
| ePRO daily diary | Item response category [1] | | | | | | | | | |
|  | Morning diary | | | | | Afternoon diary | | | | |
|  | 0 | 1 | 2 | 3 | 4 | 0 | 1 | 2 | 3 | 4 |
| 1. How hard was it to breathe air deep into your chest? | 87 (51.5%) | 40 (23.7%) | 22 (13.0%) | 10 (5.9%) | 10 (5.9%) | 79 (50.6%) | 34 (21.8%) | 21 (13.5%) | 12 (7.7%) | 10 (6.4%) |
| 2. How tight did your chest feel? | 92 (54.4%) | 28 (16.6%) | 25 (14.8%) | 14 (8.3%) | 10 (5.9%) | 82 (52.6%) | 35 (22.4%) | 15 (9.6%) | 14 (9.0%) | 10 (6.4%) |
| 3. How much has your chest hurt when you’ve coughed? | 86 (50.9%) | 33 (19.5%) | 19 (11.2%) | 18 (10.7%) | 13 (7.7%) | 79 (50.6%) | 35 (22.4%) | 16 (10.3%) | 12 (7.7%) | 14 (9.0%) |
| 4. How heavy did your chest feel? | 95 (56.2%) | 31 (18.3%) | 20 (11.8%) | 14 (8.3%) | 9 (5.3%) | 86 (55.1%) | 29 (18.6%) | 21 (13.5%) | 13 (8.3%) | 7 (4.5%) |
| 5. How much did your chest feel full of mucus (goo)? | 85 (50.3%) | 36 (21.3%) | 19 (11.2%) | 12 (7.1%) | 17 (10.1%) | 79 (50.6%) | 34 (21.8%) | 20 (12.8%) | 13 (8.3%) | 10 (6.4%) |
| 6. How stuffed up did your chest feel? | 84 (49.7%) | 40 (23.7%) | 16 (9.5%) | 14 (8.3%) | 15 (8.9%) | 78 (50.0%) | 34 (21.8%) | 20 (12.8%) | 11 (7.1%) | 13 (8.3%) |
| 7. How clogged up did your chest feel? | 90 (53.3%) | 31 (18.3%) | 17 (10.1%) | 16 (9.5%) | 15 (8.9%) | 81 (51.9%) | 32 (20.5%) | 20 (12.8%) | 13 (8.3%) | 10 (6.4%) |
| 8. How hard was it to clear your chest? | 87 (51.5%) | 38 (22.5%) | 21 (12.4%) | 12 (7.1%) | 11 (6.5%) | 80 (51.3%) | 30 (19.2%) | 25 (16.0%) | 9 (5.8%) | 12 (7.7%) |
| 9. How hard was it to clear your throat? | 86 (50.9%) | 32 (18.9%) | 20 (11.8%) | 13 (7.7%) | 18 (10.7%) | 73 (46.8%) | 37 (23.7%) | 20 (12.8%) | 12 (7.7%) | 14 (9.0%) |
| 10. How hard was it to cough up mucus (goo) from your chest? | 86 (50.9%) | 31 (18.3%) | 21 (12.4%) | 12 (7.1%) | 19 (11.2%) | 80 (51.3%) | 31 (19.9%) | 17 (10.9%) | 13 (8.3%) | 15 (9.6%) |
| *[1] Missing data excluded from this analysis so that the percentages are calculated based on those patients who completed the item.* | | | | | | | | | | |

| Supplementary table 4. Item distribution: chest-related ePRO morning/afternoon diary for participants aged 6-8 years at Day 1 (n=42) | | | | | | | | | | |
| --- | --- | --- | --- | --- | --- | --- | --- | --- | --- | --- |
| ePRO daily diary | Item response category [1] | | | | | | | | | |
|  | Morning diary | | | | | Afternoon diary | | | | |
|  | 0 | 1 | 2 | 3 | 4 | 0 | 1 | 2 | 3 | 4 |
| 1. How hard was it to breathe air deep into your chest? | 3  (7.5%) | 4 (10.0%) | 11 (27.5%) | 11 (27.5%) | 11 (27.5%) | 7 (17.9%) | 4 (10.3%) | 8 (20.5%) | 13 (33.3%) | 7 (17.9%) |
| 2. How tight did your chest feel? | 2 (5.0%) | 4 (10.0%) | 9 (22.5%) | 14 (35.0%) | 11 (27.5%) | 6 (15.4%) | 1 (2.6%) | 13 (33.3%) | 12 (30.8%) | 7 (17.9%) |
| 3. How much has your chest hurt when you’ve coughed? | 4 (10.0%) | 2 (5.0%) | 4 (10.0%) | 14 (35.0%) | 16 (40.0%) | 5 (12.8%) | 2 (5.1%) | 5 (12.8%) | 12 (30.8%) | 15 (38.5%) |
| 4. How heavy did your chest feel? | 4 (10.0%) | 4 (10.0%) | 10 (25.0%) | 14 (35.0%) | 8 (20.0%) | 7 (17.9%) | 1 (2.6%) | 9 (23.1%) | 13 (33.3%) | 9 (23.1%) |
| 5. How much did your chest feel full of mucus (goo)? | 5 (12.5%) | 7 (17.5%) | 11 (27.5%) | 17 (42.5%) | - | 3 (7.7%) | 4 (10.3%) | 4 (10.3%) | 13 (33.3%) | 15 (38.5%) |
| 6. How stuffed up did your chest feel? | 2 (5.0%) | 4 (10.0%) | 7 (17.5%) | 15 (37.5%) | 12 (30.0%) | 7 (17.9%) | - | 8 (20.5%) | 13 (33.3%) | 11 (28.2%) |
| 7. How clogged up did your chest feel? | 2 (5.0%) | 2 (5.0%) | 10 (25.0%) | 15 (37.5%) | 11 (27.5%) | 5 (12.8%) | 2 (5.1%) | 4 (10.3%) | 16 (41.0%) | 12 (30.8%) |
| 8. How hard was it to clear your chest? | 4 (10.0%) | 3 (7.5%) | 10 (25.0%) | 9 (22.5%) | 14 (35.0%) | 2 (5.1%) | 2 (5.1%) | 10 (25.6%) | 12 (30.8%) | 13 (33.3%) |
| 9. How hard was it to clear your throat? | 8 (20.0%) | 3 (7.5%) | 6 (15.0%) | 12 (30.0%) | 11 (27.5%) | 5 (12.8%) | 3 (7.7%) | 5 (12.8%) | 13 (33.3%) | 13 (33.3%) |
| 10. How hard was it to cough up mucus (goo) from your chest? | 5 (12.5%) | 3 (7.5%) | 4 (10.0%) | 14 (35.0%) | 14 (35.0%) | 5 (12.8%) | - | 8 (20.5%) | 12 (30.8%) | 14 (35.9%) |
| *[1] Missing data excluded from this analysis so that the percentages are calculated based on those patients who completed the item.* | | | | | | | | | | |

| Supplementary table 5. Item distribution: chest-related ePRO morning/afternoon diary for participants aged 6-8 years at Day 5 (n=39) | | | | | | | | | | |
| --- | --- | --- | --- | --- | --- | --- | --- | --- | --- | --- |
| ePRO daily diary | Item response category [1] | | | | | | | | | |
|  | Morning diary | | | | | Afternoon diary | | | | |
|  | 0 | 1 | 2 | 3 | 4 | 0 | 1 | 2 | 3 | 4 |
| 1. How hard was it to breathe air deep into your chest? | 11 (29.7%) | 4 (10.8%) | 15 (40.5%) | 1 (2.7%) | 6 (16.2%) | 15 (38.5%) | 4 (10.3%) | 11 (28.2%) | 5 (12.8%) | 4 (10.3%) |
| 2. How tight did your chest feel? | 10 (27.0%) | 5 (13.5%) | 12 (32.4%) | 4 (10.8%) | 6 (16.2%) | 10 (25.6%) | 6 (15.4%) | 14 (35.9%) | 4 (10.3%) | 5 (12.8%) |
| 3. How much has your chest hurt when you’ve coughed? | 8 (21.6%) | 7 (18.9%) | 9 (24.3%) | 6 (16.2%) | 7 (18.9%) | 12 (30.8%) | 6 (15.4%) | 9 (23.1%) | 6 (15.4%) | 6 (15.4%) |
| 4. How heavy did your chest feel? | 11 (29.7%) | 8 (21.6%) | 10 (27.0%) | 3 (8.1%) | 5 (13.5%) | 15 (38.5%) | 7 (17.9%) | 8 (20.5%) | 4 (10.3%) | 5 (12.8%) |
| 5. How much did your chest feel full of mucus (goo)? | 8 (21.6%) | 10 (27.0%) | 9 (24.3%) | 4 (10.8%) | 6 (16.2%) | 9 (23.1%) | 8 (20.5%) | 12 (30.8%) | 6 (15.4%) | 4 (10.3%) |
| 6. How stuffed up did your chest feel? | 8 (21.6%) | 9 (24.3%) | 10 (27.0%) | 4 (10.8%) | 6 (16.2%) | 11 (28.2%) | 4 (10.3%) | 14 (35.9%) | 5 (12.8%) | 5 (12.8%) |
| 7. How clogged up did your chest feel? | 8 (21.6%) | 8 (21.6%) | 11 (29.7%) | 3 (8.1%) | 7 (18.9%) | 10 (25.6%) | 7 (17.9%) | 8 (20.5%) | 8 (20.5%) | 6 (15.4%) |
| 8. How hard was it to clear your chest? | 9 (24.3%) | 4 (10.8%) | 14 (37.8%) | 3 (8.1%) | 7 (18.9%) | 10 (25.6%) | 5 (12.8%) | 11 (28.2%) | 5 (12.8%) | 8 (20.5%) |
| 9. How hard was it to clear your throat? | 9 (24.3%) | 6 (16.2%) | 9 (24.3%) | 4 (10.8%) | 9 (24.3%) | 12 (30.8%) | 3 (7.7%) | 10 (25.6%) | 5 (12.8%) | 9 (23.1%) |
| 10. How hard was it to cough up mucus (goo) from your chest? | 9 (24.3%) | 5 (13.5%) | 10 (27.0%) | 4 (10.8%) | 9 (24.3%) | 10 (25.6%) | 2 (5.1%) | 12 (30.8%) | 7 (17.9%) | 8 (20.5%) |
| *[1] Missing data excluded from this analysis so that the percentages are calculated based on those patients who completed the item.* | | | | | | | | | | |

| Supplementary table 6. Item distribution: chest-related ePRO morning/afternoon diary for participants aged 6-8 years at Day 10 (n=34) | | | | | | | | | | |
| --- | --- | --- | --- | --- | --- | --- | --- | --- | --- | --- |
| ePRO daily diary | Item response category [1] | | | | | | | | | |
|  | Morning diary | | | | | Afternoon diary | | | | |
|  | 0 | 1 | 2 | 3 | 4 | 0 | 1 | 2 | 3 | 4 |
| 1. How hard was it to breathe air deep into your chest? | 15 (48.4%) | 9 (29.0%) | 4 (12.9%) | 2 (6.5%) | 1 (3.2%) | 12 (41.4%) | 8 (27.6%) | 4 (13.8%) | 3 (10.3%) | 2 (6.9%) |
| 2. How tight did your chest feel? | 17 (54.8%) | 4 (12.9%) | 3 (9.7%) | 5 (16.1%) | 2 (6.5%) | 14 (48.3%) | 8 (27.6%) | 1 (3.4%) | 5 (17.2%) | 1 (3.4%) |
| 3. How much has your chest hurt when you’ve coughed? | 15 (48.4%) | 6 (19.4%) | 3 (9.7%) | 5 (16.1%) | 2 (6.5%) | 14 (48.3%) | 7 (24.1%) | 2 (6.9%) | 3 (10.3%) | 3 (10.3%) |
| 4. How heavy did your chest feel? | 17 (54.8%) | 7 (22.6%) | 2 (6.5%) | 4 (12.9%) | 1 (3.2%) | 16 (55.2%) | 5 (17.2%) | 4 (13.8%) | 3 (10.3%) | 1 (3.4%) |
| 5. How much did your chest feel full of mucus (goo)? | 13 (41.9%) | 6 (19.4%) | 6 (19.4%) | 3 (9.7%) | 3 (9.7%) | 12 (41.4%) | 8 (27.6%) | 3 (10.3%) | 5 (17.2%) | 1 (3.4%) |
| 6. How stuffed up did your chest feel? | 13 (41.9%) | 9 (29.0%) | 2 (6.5%) | 3 (9.7%) | 4 (12.9%) | 13 (44.8%) | 7 (24.1%) | 5 (17.2%) | 2 (6.9%) | 2 (6.9%) |
| 7. How clogged up did your chest feel? | 15 (48.4%) | 7 (22.6%) | 3 (9.7%) | 3 (9.7%) | 3 (9.7%) | 15 (51.7%) | 6 (20.7%) | 4 (13.8%) | 3 (10.3%) | 1 (3.4%) |
| 8. How hard was it to clear your chest? | 14 (45.2%) | 8 (25.8%) | 4 (12.9%) | 3 (9.7%) | 2 (6.5%) | 14 (48.3%) | 7 (24.1%) | 3 (10.3%) | 3 (10.3%) | 2 (6.9%) |
| 9. How hard was it to clear your throat? | 11 (35.5%) | 8 (25.8%) | 5 (16.1%) | 3 (9.7%) | 4 (12.9%) | 14 (48.3%) | 4 (13.8%) | 4 (13.8%) | 3 (10.3%) | 4 (13.8%) |
| 10. How hard was it to cough up mucus (goo) from your chest? | 13 (41.9%) | 7 (22.6%) | 6 (19.4%) | 1 (3.2%) | 4 (12.9%) | 14 (48.3%) | 8 (27.6%) | 2 (6.9%) | 2 (6.9%) | 3 (10.3%) |
| *[1] Missing data excluded from this analysis so that the percentages are calculated based on those patients who completed the item.* | | | | | | | | | | |

| Supplementary table 7. Item distribution: chest-related ePRO morning/afternoon diary for participants aged 9-11 years at Day 1 (n=46) | | | | | | | | | | |
| --- | --- | --- | --- | --- | --- | --- | --- | --- | --- | --- |
| ePRO daily diary | Item response category [1] | | | | | | | | | |
|  | Morning diary | | | | | Afternoon diary | | | | |
|  | 0 | 1 | 2 | 3 | 4 | 0 | 1 | 2 | 3 | 4 |
| 1. How hard was it to breathe air deep into your chest? | 3 (7.0%) | 2 (4.7%) | 13 (30.2%) | 14 (32.6%) | 11 (25.6%) | 2 (4.8%) | 10 (23.8%) | 13 (31.0%) | 11 (26.2%) | 6 (14.3%) |
| 2. How tight did your chest feel? | 1 (2.3%) | 5 (11.6%) | 17 (39.5%) | 4 (9.3%) | 16 (37.2%) | 1 (2.4%) | 10 (23.8%) | 7 (16.7%) | 15 (35.7%) | 9 (21.4%) |
| 3. How much has your chest hurt when you’ve coughed? | 4 (9.3%) | 7 (16.3%) | 13 (30.2%) | 19 (44.2%) | - | 2 (4.8%) | 9 (21.4%) | 3 (7.1%) | 12 (28.6%) | 16 (38.1%) |
| 4. How heavy did your chest feel? | 1 (2.3%) | 4 (9.3%) | 15 (34.9%) | 13 (30.2%) | 10 (23.3%) | 1 (2.4%) | 6 (14.3%) | 11 (26.2%) | 16 (38.1%) | 8 (19.0%) |
| 5. How much did your chest feel full of mucus (goo)? | 3 (7.0%) | 6 (14.0%) | 14 (32.6%) | 20 (46.5%) | - | 1 (2.4%) | 7 (16.7%) | 9 (21.4%) | 11 (26.2%) | 14 (33.3%) |
| 6. How stuffed up did your chest feel? | 1 (2.3%) | 3 (7.0%) | 8 (18.6%) | 15 (34.9%) | 16 (37.2%) | 7 (16.7%) | 7 (16.7%) | 17 (40.5%) | 11 (26.2%) | - |
| 7. How clogged up did your chest feel? | 1 (2.3%) | 3 (7.0%) | 8 (18.6%) | 14 (32.6%) | 17 (39.5%) | 1 (2.4%) | 7 (16.7%) | 6 (14.3%) | 17 (40.5%) | 11 (26.2%) |
| 8. How hard was it to clear your chest? | 1 (2.3%) | 1 (2.3%) | 13 (30.2%) | 11 (25.6%) | 17 (39.5%) | 5 (11.9%) | 10 (23.8%) | 16 (38.1%) | 11 (26.2%) | - |
| 9. How hard was it to clear your throat? | 3 (7.0%) | 10 (23.3%) | 12 (27.9%) | 18 (41.9%) | - | 1 (2.4%) | 5 (11.9%) | 11 (26.2%) | 13 (31.0%) | 12 (28.6%) |
| 10. How hard was it to cough up mucus (goo) from your chest? | 2 (4.7%) | 2 (4.7%) | 9 (20.9%) | 12 (27.9%) | 18 (41.9%) | 1 (2.4%) | 3 (7.1%) | 11 (26.2%) | 14 (33.3%) | 13 (31.0%) |
| *[1] Missing data excluded from this analysis so that the percentages are calculated based on those patients who completed the item.* | | | | | | | | | | |

| Supplementary table 8. Item distribution: chest-related ePRO morning/afternoon diary for participants aged 9-11 years at Day 5 (n=45) | | | | | | | | | | |
| --- | --- | --- | --- | --- | --- | --- | --- | --- | --- | --- |
| ePRO daily diary | Item response category [1] | | | | | | | | | |
|  | Morning diary | | | | | Afternoon diary | | | | |
|  | 0 | 1 | 2 | 3 | 4 | 0 | 1 | 2 | 3 | 4 |
| 1. How hard was it to breathe air deep into your chest? | 9 (20.5%) | 12 (27.3%) | 13 (29.5%) | 7 (15.9%) | 3 (6.8%) | 7 (17.1%) | 7 (17.1%) | 14 (34.1%) | 7 (17.1%) | 6 (14.6%) |
| 2. How tight did your chest feel? | 7 (15.9%) | 15 (34.1%) | 8 (18.2%) | 8 (18.2%) | 6 (13.6%) | 6 (14.6%) | 8 (19.5%) | 10 (24.4%) | 10 (24.4%) | 7 (17.1%) |
| 3. How much has your chest hurt when you’ve coughed? | 9 (20.5%) | 6 (13.6%) | 15 (34.1%) | 8 (18.2%) | 6 (13.6%) | 6 (14.6%) | 13 (31.7%) | 7 (17.1%) | 5 (12.2%) | 10 (24.4%) |
| 4. How heavy did your chest feel? | 6 (13.6%) | 13 (29.5%) | 14 (31.8%) | 5 (11.4%) | 6 (13.6%) | 6 (14.6%) | 7 (17.1%) | 15 (36.6%) | 6 (14.6%) | 7 (17.1%) |
| 5. How much did your chest feel full of mucus (goo)? | 7 (15.9%) | 9 (20.5%) | 12 (27.3%) | 10 (22.7%) | 6 (13.6%) | 5 (12.2%) | 11 (26.8%) | 9 (22.0%) | 6 (14.6%) | 10 (24.4%) |
| 6. How stuffed up did your chest feel? | 7 (15.9%) | 10 (22.7%) | 12 (27.3%) | 9 (20.5%) | 6 (13.6%) | 3 (7.3%) | 10 (24.4%) | 14 (34.1%) | 4 (9.8%) | 10 (24.4%) |
| 7. How clogged up did your chest feel? | 8 (18.2%) | 10 (22.7%) | 11 (25.0%) | 11 (25.0%) | 4 (9.1%) | 5 (12.2%) | 6 (14.6%) | 13 (31.7%) | 7 (17.1%) | 10 (24.4%) |
| 8. How hard was it to clear your chest? | 7 (15.9%) | 5 (11.4%) | 17 (38.6%) | 7 (15.9%) | 8 (18.2%) | 4 (9.8%) | 7 (17.1%) | 14 (34.1%) | 6 (14.6%) | 10 (24.4%) |
| 9. How hard was it to clear your throat? | 6 (13.6%) | 8 (18.2%) | 12 (27.3%) | 10 (22.7%) | 8 (18.2%) | 3 (7.3%) | 8 (19.5%) | 12 (29.3%) | 8 (19.5%) | 10 (24.4%) |
| 10. How hard was it to cough up mucus (goo) from your chest? | 7 (15.9%) | 13 (29.5%) | 9 (20.5%) | 7 (15.9%) | 8 (18.2%) | 5 (12.2%) | 8 (19.5%) | 12 (29.3%) | 5 (12.2%) | 11 (26.8%) |
| *[1] Missing data excluded from this analysis so that the percentages are calculated based on those patients who completed the item.* | | | | | | | | | | |

| Supplementary table 9. Item distribution: chest-related ePRO morning/afternoon diary for participants aged 9-11 years at Day 10 (n=40) | | | | | | | | | | |
| --- | --- | --- | --- | --- | --- | --- | --- | --- | --- | --- |
| ePRO daily diary | Item response category [1] | | | | | | | | | |
|  | Morning diary | | | | | Afternoon diary | | | | |
|  | 0 | 1 | 2 | 3 | 4 | 0 | 1 | 2 | 3 | 4 |
| 1. How hard was it to breathe air deep into your chest? | 15 (39.5%) | 9 (23.7%) | 7 (18.4%) | 3 (7.9%) | 4 (10.5%) | 14 (36.8%) | 6 (15.8%) | 8 (21.1%) | 4 (10.5%) | 6 (15.8%) |
| 2. How tight did your chest feel? | 18 (47.4%) | 6 (15.8%) | 8 (21.1%) | 3 (7.9%) | 3 (7.9%) | 13 (34.2%) | 6 (15.8%) | 7 (18.4%) | 7 (18.4%) | 5 (13.2%) |
| 3. How much has your chest hurt when you’ve coughed? | 16 (42.1%) | 7 (18.4%) | 5 (13.2%) | 4 (10.5%) | 6 (15.8%) | 13 (34.2%) | 5 (13.2%) | 7 (18.4%) | 6 (15.8%) | 7 (18.4%) |
| 4. How heavy did your chest feel? | 17 (44.7%) | 7 (18.4%) | 5 (13.2%) | 5 (13.2%) | 4 (10.5%) | 12 (31.6%) | 10 (26.3%) | 8 (21.1%) | 4 (10.5%) | 4 (10.5%) |
| 5. How much did your chest feel full of mucus (goo)? | 16 (42.1%) | 10 (26.3%) | 3 (7.9%) | 1 (2.6%) | 8 (21.1%) | 11 (28.9%) | 9 (23.7%) | 4 (10.5%) | 7 (18.4%) | 7 (18.4%) |
| 6. How stuffed up did your chest feel? | 15 (39.5%) | 8 (21.1%) | 6 (15.8%) | 3 (7.9%) | 6 (15.8%) | 11 (28.9%) | 6 (15.8%) | 9 (23.7%) | 5 (13.2%) | 7 (18.4%) |
| 7. How clogged up did your chest feel? | 15 (39.5%) | 8 (21.1%) | 5 (13.2%) | 3 (7.9%) | 7 (18.4%) | 14 (36.8%) | 7 (18.4%) | 4 (10.5%) | 7 (18.4%) | 6 (15.8%) |
| 8. How hard was it to clear your chest? | 15 (39.5%) | 7 (18.4%) | 7 (18.4%) | 5 (13.2%) | 4 (10.5%) | 11 (28.9%) | 6 (15.8%) | 10 (26.3%) | 4 (10.5%) | 7 (18.4%) |
| 9. How hard was it to clear your throat? | 16 (42.1%) | 5 (13.2%) | 5 (13.2%) | 4 (10.5%) | 8 (21.1%) | 11 (28.9%) | 3 (7.9%) | 8 (21.1%) | 7 (18.4%) | 9 (23.7%) |
| 10. How hard was it to cough up mucus (goo) from your chest? | 17 (44.7%) | 6 (15.8%) | 4 (10.5%) | 3 (7.9%) | 8 (21.1%) | 15 (39.5%) | 6 (15.8%) | 4 (10.5%) | 5 (13.2%) | 8 (21.1%) |
| *[1] Missing data excluded from this analysis so that the percentages are calculated based on those patients who completed the item.* | | | | | | | | | | |

| Supplementary table 10. Item distribution: chest-related ePRO morning/afternoon diary for participants aged 12-17 years at Day 1 (n=55) | | | | | | | | | | |
| --- | --- | --- | --- | --- | --- | --- | --- | --- | --- | --- |
| ePRO daily diary | Item response category [1] | | | | | | | | | |
|  | Morning diary | | | | | Afternoon diary | | | | |
|  | 0 | 1 | 2 | 3 | 4 | 0 | 1 | 2 | 3 | 4 |
| 1. How hard was it to breathe air deep into your chest? | 3 (5.5%) | 5 (9.1%) | 10 (18.2%) | 20 (36.4%) | 17 (30.9%) | 4 (7.7%) | 5 (9.6%) | 12 (23.1%) | 13 (25.0%) | 18 (34.6%) |
| 2. How tight did your chest feel? | 2 (3.6%) | 8 (14.5%) | 9 (16.4%) | 18 (32.7%) | 18 (32.7%) | 4 (7.7%) | 7 (13.5%) | 7 (13.5%) | 20 (38.5%) | 14 (26.9%) |
| 3. How much has your chest hurt when you’ve coughed? | 3 (5.5%) | 5 (9.1%) | 8 (14.5%) | 14 (25.5%) | 25 (45.5%) | 3 (5.8%) | 5 (9.6%) | 5 (9.6%) | 12 (23.1%) | 27 (51.9%) |
| 4. How heavy did your chest feel? | 2 (3.6%) | 7 (12.7%) | 9 (16.4%) | 21 (38.2%) | 16 (29.1%) | 2 (3.8%) | 8 (15.4%) | 12 (23.1%) | 15 (28.8%) | 15 (28.8%) |
| 5. How much did your chest feel full of mucus (goo)? | 2 (3.6%) | 5 (9.1%) | 5 (9.1%) | 18 (32.7%) | 25 (45.5%) | 2 (3.8%) | 7 (13.5%) | 7 (13.5%) | 12 (23.1%) | 24 (46.2%) |
| 6. How stuffed up did your chest feel? | 3 (5.5%) | 4 (7.3%) | 8 (14.5%) | 19 (34.5%) | 21 (38.2%) | 1 (1.9%) | 4 (7.7%) | 12 (23.1%) | 13 (25.0%) | 22 (42.3%) |
| 7. How clogged up did your chest feel? | 2 (3.6%) | 6 (10.9%) | 7 (12.7%) | 14 (25.5%) | 26 (47.3%) | 4 (7.7%) | 4 (7.7%) | 11 (21.2%) | 11 (21.2%) | 22 (42.3%) |
| 8. How hard was it to clear your chest? | 3 (5.5%) | 7 (12.7%) | 8 (14.5%) | 13 (23.6%) | 24 (43.6%) | 2 (3.8%) | 4 (7.7%) | 11 (21.2%) | 13 (25.0%) | 22 (42.3%) |
| 9. How hard was it to clear your throat? | 2 (3.6%) | 2 (3.6%) | 12 (21.8%) | 15 (27.3%) | 24 (43.6%) | 2 (3.8%) | 3 (5.8%) | 6 (11.5%) | 16 (30.8%) | 25 (48.1%) |
| 10. How hard was it to cough up mucus (goo) from your chest? | 2 (3.6%) | 2 (3.6%) | 8 (14.5%) | 19 (34.5%) | 24 (43.6%) | 1 (1.9%) | 6 (11.5%) | 5 (9.6%) | 13 (25.0%) | 27 (51.9%) |
| *[1] Missing data excluded from this analysis so that the percentages are calculated based on those patients who completed the item.* | | | | | | | | | | |

| Supplementary table 11. Item distribution: chest-related ePRO morning/afternoon diary for participants aged 12-17 years at Day 5 (n=53) | | | | | | | | | | |
| --- | --- | --- | --- | --- | --- | --- | --- | --- | --- | --- |
| ePRO daily diary | Item response category [1] | | | | | | | | | |
|  | Morning diary | | | | | Afternoon diary | | | | |
|  | 0 | 1 | 2 | 3 | 4 | 0 | 1 | 2 | 3 | 4 |
| 1. How hard was it to breathe air deep into your chest? | 11 (21.2%) | 9 (17.3%) | 12 (23.1%) | 11 (21.2%) | 9 (17.3%) | 9 (17.6%) | 13 (25.5%) | 13 (25.5%) | 7 (13.7%) | 9 (17.6%) |
| 2. How tight did your chest feel? | 10 (19.2%) | 9 (17.3%) | 13 (25.0%) | 11 (21.2%) | 9 (17.3%) | 8 (15.7%) | 12 (23.5%) | 14 (27.5%) | 10 (19.6%) | 7 (13.7%) |
| 3. How much has your chest hurt when you’ve coughed? | 6 (11.5%) | 11 (21.2%) | 17 (32.7%) | 8 (15.4%) | 10 (19.2%) | 8 (15.7%) | 14 (27.5%) | 7 (13.7%) | 13 (25.5%) | 9 (17.6%) |
| 4. How heavy did your chest feel? | 9 (17.3%) | 12 (23.1%) | 12 (23.1%) | 10 (19.2%) | 9 (17.3%) | 8 (15.7%) | 13 (25.5%) | 13 (25.5%) | 9 (17.6%) | 8 (15.7%) |
| 5. How much did your chest feel full of mucus (goo)? | 8 (15.4%) | 12 (23.1%) | 7 (13.5%) | 16 (30.8%) | 9 (17.3%) | 6 (11.8%) | 12 (23.5%) | 12 (23.5%) | 11 (21.6%) | 10 (19.6%) |
| 6. How stuffed up did your chest feel? | 9 (17.3%) | 12 (23.1%) | 8 (15.4%) | 12 (23.1%) | 11 (21.2%) | 5 (9.8%) | 14 (27.5%) | 10 (19.6%) | 11 (21.6%) | 11 (21.6%) |
| 7. How clogged up did your chest feel? | 8 (15.4%) | 14 (26.9%) | 7 (13.5%) | 12 (23.1%) | 11 (21.2%) | 6 (11.8%) | 12 (23.5%) | 10 (19.6%) | 12 (23.5%) | 11 (21.6%) |
| 8. How hard was it to clear your chest? | 6 (11.5%) | 12 (23.1%) | 12 (23.1%) | 12 (23.1%) | 10 (19.2%) | 9 (17.6%) | 8 (15.7%) | 13 (25.5%) | 10 (19.6%) | 11 (21.6%) |
| 9. How hard was it to clear your throat? | 8 (15.4%) | 7 (13.5%) | 15 (28.8%) | 10 (19.2%) | 12 (23.1%) | 6 (11.8%) | 12 (23.5%) | 10 (19.6%) | 12 (23.5%) | 11 (21.6%) |
| 10. How hard was it to cough up mucus (goo) from your chest? | 7 (13.5%) | 12 (23.1%) | 8 (15.4%) | 13 (25.0%) | 12 (23.1%) | 7 (13.7%) | 7 (13.7%) | 14 (27.5%) | 12 (23.5%) | 11 (21.6%) |
| *[1] Missing data excluded from this analysis so that the percentages are calculated based on those patients who completed the item.* | | | | | | | | | | |

| Supplementary table 12. Item distribution: chest-related ePRO morning/afternoon diary for participants aged 12-17 years at Day 10 (n=53) | | | | | | | | | | |
| --- | --- | --- | --- | --- | --- | --- | --- | --- | --- | --- |
| ePRO daily diary | Item response category [1] | | | | | | | | | |
|  | Morning diary | | | | | Afternoon diary | | | | |
|  | 0 | 1 | 2 | 3 | 4 | 0 | 1 | 2 | 3 | 4 |
| 1. How hard was it to breathe air deep into your chest? | 25 (48.1%) | 11 (21.2%) | 7 (13.5%) | 4 (7.7%) | 5 (9.6%) | 26 (55.3%) | 7 (14.9%) | 6 (12.8%) | 4 (8.5%) | 4 (8.5%) |
| 2. How tight did your chest feel? | 27 (51.9%) | 8 (15.4%) | 8 (15.4%) | 4 (7.7%) | 5 (9.6%) | 24 (51.1%) | 9 (19.1%) | 7 (14.9%) | 3 (6.4%) | 4 (8.5%) |
| 3. How much has your chest hurt when you’ve coughed? | 23 (44.2%) | 11 (21.2%) | 9 (17.3%) | 4 (7.7%) | 5 (9.6%) | 26 (55.3%) | 8 (17.0%) | 6 (12.8%) | 3 (6.4%) | 4 (8.5%) |
| 4. How heavy did your chest feel? | 28 (53.8%) | 7 (13.5%) | 8 (15.4%) | 5 (9.6%) | 4 (7.7%) | 28 (59.6%) | 6 (12.8%) | 8 (17.0%) | 2 (4.3%) | 3 (6.4%) |
| 5. How much did your chest feel full of mucus (goo)? | 25 (48.1%) | 10 (19.2%) | 6 (11.5%) | 6 (11.5%) | 5 (9.6%) | 29 (61.7%) | 5 (10.6%) | 6 (12.8%) | 3 (6.4%) | 4 (8.5%) |
| 6. How stuffed up did your chest feel? | 26 (50.0%) | 10 (19.2%) | 5 (9.6%) | 6 (11.5%) | 5 (9.6%) | 26 (55.3%) | 6 (12.8%) | 6 (12.8%) | 4 (8.5%) | 5 (10.6%) |
| 7. How clogged up did your chest feel? | 27 (51.9%) | 7 (13.5%) | 7 (13.5%) | 7 (13.5%) | 4 (7.7%) | 27 (57.4%) | 6 (12.8%) | 6 (12.8%) | 4 (8.5%) | 4 (8.5%) |
| 8. How hard was it to clear your chest? | 28 (53.8%) | 10 (19.2%) | 6 (11.5%) | 4 (7.7%) | 4 (7.7%) | 27 (57.4%) | 7 (14.9%) | 7 (14.9%) | 2 (4.3%) | 4 (8.5%) |
| 9. How hard was it to clear your throat? | 26 (50.0%) | 10 (19.2%) | 6 (11.5%) | 5 (9.6%) | 5 (9.6%) | 23 (48.9%) | 12 (25.5%) | 5 (10.6%) | 3 (6.4%) | 4 (8.5%) |
| 10. How hard was it to cough up mucus (goo) from your chest? | 26 (50.0%) | 6 (11.5%) | 6 (11.5%) | 7 (13.5%) | 7 (13.5%) | 25 (53.2%) | 7 (14.9%) | 5 (10.6%) | 5 (10.6%) | 5 (10.6%) |
| *[1] Missing data excluded from this analysis so that the percentages are calculated based on those patients who completed the item.* | | | | | | | | | | |

| Supplementary table 13. Item distribution: chest-related ePRO morning/afternoon diary for adult participants at Day 1 (n=48) | | | | | | | | | | |
| --- | --- | --- | --- | --- | --- | --- | --- | --- | --- | --- |
| ePRO daily diary | Item response category [1] | | | | | | | | | |
|  | Morning diary | | | | | Afternoon diary | | | | |
|  | 0 | 1 | 2 | 3 | 4 | 0 | 1 | 2 | 3 | 4 |
| 1. How hard was it to breathe air deep into your chest? | 2  (4.3%) | 13 (27.7%) | 23 (48.9%) | 9 (19.1%) | - | 1 (2.1%) | 4 (8.5%) | 12 (25.5%) | 23 (48.9%) | 7 (14.9%) |
| 2. How tight did your chest feel? | 1 (2.1%) | 16 (34.0%) | 21 (44.7%) | 9 (19.1%) | - | 1 (2.1%) | 3 (6.4%) | 18 (38.3%) | 19 (40.4%) | 6 (12.8%) |
| 3. How much has your chest hurt when you’ve coughed? | 2 (4.3%) | 12 (25.5%) | 15 (31.9%) | 18 (38.3%) | - | 1 (2.1%) | 2 (4.3%) | 14 (29.8%) | 19 (40.4%) | 11 (23.4%) |
| 4. How heavy did your chest feel? | 3  (6.4%) | 13 (27.7%) | 22 (46.8%) | 9 (19.1%) | - | 1 (2.1%) | 5 (10.6%) | 17 (36.2%) | 16 (34.0%) | 8 (17.0%) |
| 5. How much did your chest feel full of mucus (goo)? | 4 (8.5%) | 8 (17.0%) | 18 (38.3%) | 17 (36.2%) | - | 1 (2.1%) | 4 (8.5%) | 12 (25.5%) | 17 (36.2%) | 13 (27.7%) |
| 6. How stuffed up did your chest feel? | 2 (4.3%) | 9 (19.1%) | 19 (40.4%) | 17 (36.2%) | - | 1 (2.1%) | 2 (4.3%) | 14 (29.8%) | 16 (34.0%) | 14 (29.8%) |
| 7. How clogged up did your chest feel? | 2 (4.3%) | 12 (25.5%) | 18 (38.3%) | 15 (31.9%) | - | 1 (2.1%) | 2 (4.3%) | 13 (27.7%) | 18 (38.3%) | 13 (27.7%) |
| 8. How hard was it to clear your chest? | 2 (4.3%) | 16 (34.0%) | 14 (29.8%) | 15 (31.9%) | - | 1 (2.1%) | 6 (12.8%) | 12 (25.5%) | 15 (31.9%) | 13 (27.7%) |
| 9. How hard was it to clear your throat? | 1 (2.1%) | 17 (36.2%) | 15 (31.9%) | 14 (29.8%) | - | 1 (2.1%) | 4 (8.5%) | 11 (23.4%) | 21 (44.7%) | 10 (21.3%) |
| 10. How hard was it to cough up mucus (goo) from your chest? | 1 (2.1%) | 2 (4.3%) | 15 (31.9%) | 12 (25.5%) | 17 (36.2%) | 8 (17.0%) | 11 (23.4%) | 14 (29.8%) | 14 (29.8%) | - |
| *[1] Missing data excluded from this analysis so that the percentages are calculated based on those patients who completed the item.* | | | | | | | | | | |

| Supplementary table 14. Item distribution: chest-related ePRO morning/afternoon diary for adult participants at Day 5 (n=50) | | | | | | | | | | |
| --- | --- | --- | --- | --- | --- | --- | --- | --- | --- | --- |
| ePRO daily diary | Item response category [1] | | | | | | | | | |
|  | Morning diary | | | | | Afternoon diary | | | | |
|  | 0 | 1 | 2 | 3 | 4 | 0 | 1 | 2 | 3 | 4 |
| 1. How hard was it to breathe air deep into your chest? | 9 (18.0%) | 15 (30.0%) | 17 (34.0%) | 6 (12.0%) | 3 (6.0%) | 12 (26.1%) | 15 (32.6%) | 14 (30.4%) | 3 (6.5%) | 2 (4.3%) |
| 2. How tight did your chest feel? | 11 (22.0%) | 13 (26.0%) | 15 (30.0%) | 9 (18.0%) | 2 (4.0%) | 10 (21.7%) | 17 (37.0%) | 13 (28.3%) | 4 (8.7%) | 2 (4.3%) |
| 3. How much has your chest hurt when you’ve coughed? | 11 (22.0%) | 14 (28.0%) | 15 (30.0%) | 8 (16.0%) | 2 (4.0%) | 13 (28.3%) | 12 (26.1%) | 12 (26.1%) | 5 (10.9%) | 4 (8.7%) |
| 4. How heavy did your chest feel? | 13 (26.0%) | 12 (24.0%) | 16 (32.0%) | 7 (14.0%) | 2 (4.0%) | 11 (23.9%) | 16 (34.8%) | 14 (30.4%) | 3 (6.5%) | 2 (4.3%) |
| 5. How much did your chest feel full of mucus (goo)? | 10 (20.0%) | 16 (32.0%) | 15 (30.0%) | 6 (12.0%) | 3 (6.0%) | 12 (26.1%) | 15 (32.6%) | 11 (23.9%) | 3 (6.5%) | 5 (10.9%) |
| 6. How stuffed up did your chest feel? | 9 (18.0%) | 16 (32.0%) | 16 (32.0%) | 6 (12.0%) | 3 (6.0%) | 10 (21.7%) | 16 (34.8%) | 13 (28.3%) | 3 (6.5%) | 4 (8.7%) |
| 7. How clogged up did your chest feel? | 12 (24.0%) | 12 (24.0%) | 15 (30.0%) | 6 (12.0%) | 5 (10.0%) | 12 (26.1%) | 12 (26.1%) | 16 (34.8%) | 2 (4.3%) | 4 (8.7%) |
| 8. How hard was it to clear your chest? | 13 (26.0%) | 11 (22.0%) | 17 (34.0%) | 7 (14.0%) | 2 (4.0%) | 11 (23.9%) | 14 (30.4%) | 14 (30.4%) | 2 (4.3%) | 5 (10.9%) |
| 9. How hard was it to clear your throat? | 11 (22.0%) | 12 (24.0%) | 15 (30.0%) | 10 (20.0%) | 2 (4.0%) | 11 (23.9%) | 14 (30.4%) | 13 (28.3%) | 6 (13.0%) | 2 (4.3%) |
| 10. How hard was it to cough up mucus (goo) from your chest? | 11 (22.0%) | 10 (20.0%) | 13 (26.0%) | 13 (26.0%) | 3 (6.0%) | 9 (19.6%) | 14 (30.4%) | 14 (30.4%) | 4 (8.7%) | 5 (10.9%) |
| *[1] Missing data excluded from this analysis so that the percentages are calculated based on those patients who completed the item.* | | | | | | | | | | |

| Supplementary table 15. Item distribution: chest-related ePRO morning/afternoon diary for adult participants at Day 10 (n=50) | | | | | | | | | | |
| --- | --- | --- | --- | --- | --- | --- | --- | --- | --- | --- |
| ePRO daily diary | Item response category [1] | | | | | | | | | |
|  | Morning diary | | | | | Afternoon diary | | | | |
|  | 0 | 1 | 2 | 3 | 4 | 0 | 1 | 2 | 3 | 4 |
| 1. How hard was it to breathe air deep into your chest? | 32 (66.7%) | 11 (22.9%) | 4 (8.3%) | 1 (2.1%) | - | 25 (58.1%) | 13 (30.2%) | 5 (11.6%) | - | - |
| 2. How tight did your chest feel? | 30 (62.5%) | 10 (20.8%) | 6 (12.5%) | 2 (4.2%) | - | 26 (60.5%) | 11 (25.6%) | 4 (9.3%) | 2 (4.7%) | - |
| 3. How much has your chest hurt when you’ve coughed? | 32 (66.7%) | 9 (18.8%) | 2 (4.2%) | 5 (10.4%) | - | 25 (58.1%) | 11 (25.6%) | 4 (9.3%) | 3 (7.0%) | - |
| 4. How heavy did your chest feel? | 33 (68.8%) | 10 (20.8%) | 5 (10.4%) | - | - | 25 (58.1%) | 12 (27.9%) | 3 (7.0%) | 3 (7.0%) | - |
| 5. How much did your chest feel full of mucus (goo)? | 31 (64.6%) | 10 (20.8%) | 4 (8.3%) | 2 (4.2%) | 1 (2.1%) | 25 (58.1%) | 13 (30.2%) | 3 (7.0%) | 2 (4.7%) | - |
| 6. How stuffed up did your chest feel? | 30 (62.5%) | 13 (27.1%) | 3 (6.3%) | 2 (4.2%) | - | 25 (58.1%) | 14 (32.6%) | 2 (4.7%) | 2 (4.7%) | - |
| 7. How clogged up did your chest feel? | 33 (68.8%) | 9 (18.8%) | 2 (4.2%) | 3 (6.3%) | 1 (2.1%) | 24 (55.8%) | 13 (30.2%) | 4 (9.3%) | 2 (4.7%) | - |
| 8. How hard was it to clear your chest? | 30 (62.5%) | 13 (27.1%) | 4 (8.3%) | - | 1 (2.1%) | 26 (60.5%) | 10 (23.3%) | 7 (16.3%) | - | - |
| 9. How hard was it to clear your throat? | 33 (68.8%) | 9 (18.8%) | 4 (8.3%) | 1 (2.1%) | 1 (2.1%) | 24 (55.8%) | 15 (34.9%) | 4 (9.3%) | - | - |
| 10. How hard was it to cough up mucus (goo) from your chest? | 30 (62.5%) | 12 (25.0%) | 5 (10.4%) | 1 (2.1%) | - | 26 (60.5%) | 10 (23.3%) | 5 (11.6%) | 2 (4.7%) | - |
| *[1] Missing data excluded from this analysis so that the percentages are calculated based on those patients who completed the item.* | | | | | | | | | | |

## Mean chest-related ePRO diary scores


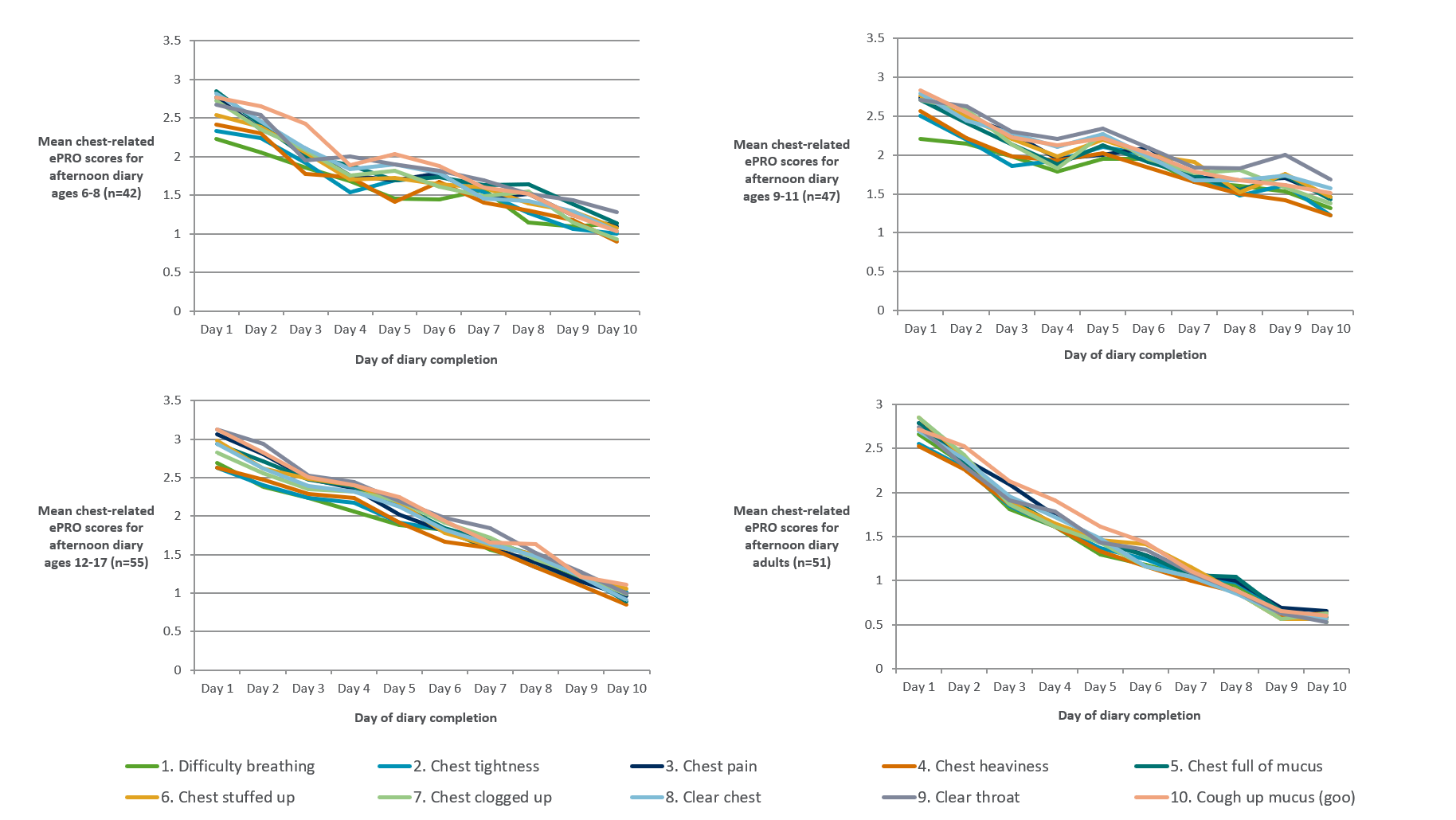


Supplementary figure 4. Mean chest-related ePRO scores for the afternoon diary across all age groups from Day 1 to Day 10

## Item performance

| Supplementary table 16. Inter-item Pearson correlations for the chest-related ePRO morning diary at Day 1 N=191; 6-8 years (n=42), 9-11 years (n=46), 12-17 years (n=55), adults (n=48) | | | | | | | | | | | |
| --- | --- | --- | --- | --- | --- | --- | --- | --- | --- | --- | --- |
|  |  | Chest-related ePRO diary items | | | | | | | | | |
| Item | Age group | 1 | 2 | 3 | 4 | 5 | 6 | 7 | 8 | 9 | 10 |
| Morning 1 (difficulty breathing) | **All ages** | - | - | - | - | - | - | - | - | - | - |
|  | **6-8 years** | - | - | - | - | - | - | - | - | - | - |
|  | **9-11 yrs** | - | - | - | - | - | - | - | - | - | - |
|  | **12-17 yrs** | - | - | - | - | - | - | - | - | - | - |
|  | **Adults** | - | - | - | - | - | - | - | - | - | - |
| Morning 2  (chest tightness) | **All ages** | 0.754 | - | - | - | - | - | - | - | - | - |
|  | **6-8 yrs** | 0.665 | - | - | - | - | - | - | - | - | - |
|  | **9-11 yrs** | 0.758 | - | - | - | - | - | - | - | - | - |
|  | **12-17 yrs** | 0.814 | - | - | - | - | - | - | - | - | - |
|  | **Adults** | 0.768 | - | - | - | - | - | - | - | - | - |
| Morning 3  (chest pain) | **All ages** | 0.613 | 0.642 | - | - | - | - | - | - | - | - |
|  | **6-8 yrs** | 0.615 | 0.526 | - | - | - | - | - | - | - | - |
|  | **9-11 yrs** | 0.473 | 0.579 | - | - | - | - | - | - | - | - |
|  | **12-17 yrs** | 0.709 | 0.813 | - | - | - | - | - | - | - | - |
|  | **Adults** | 0.617 | 0.572 | - | - | - | - | - | - | - | - |
| Morning 4  (chest heaviness) | **All ages** | 0.717 | 0.739 | 0.579 | - | - | - | - | - | - | - |
|  | **6-8 years** | 0.668 | 0.563 | 0.376 | - | - | - | - | - | - | - |
|  | **9-11 yrs** | 0.728 | 0.831 | 0.712 | - | - | - | - | - | - | - |
|  | **12-17 yrs** | 0.763 | 0.817 | 0.645 | - | - | - | - | - | - | - |
|  | **Adults** | 0.672 | 0.749 | 0.645 | - | - | - | - | - | - | - |
| Morning 5  (chest full of mucus) | **All ages** | 0.664 | 0.678 | 0.635 | 0.610 | - | - | - | - | - | - |
|  | **6-8 years** | 0.655 | 0.764 | 0.529 | 0.475 | - | - | - | - | - | - |
|  | **9-11 yrs** | 0.492 | 0.494 | 0.597 | 0.598 | - | - | - | - | - | - |
|  | **12-17 yrs** | 0.818 | 0.762 | 0.777 | 0.707 | - | - | - | - | - | - |
|  | **Adults** | 0.647 | 0.694 | 0.557 | 0.670 | - | - | - | - | - | - |
| Morning 6  (chest stuffed up) | **All ages** | 0.692 | 0.651 | 0.553 | 0.719 | 0.715 | - | - | - | - | - |
|  | **6-8 years** | 0.685 | 0.598 | 0.335 | 0.737 | 0.591 | - | - | - | - | - |
|  | **9-11 yrs** | 0.603 | 0.663 | 0.626 | 0.711 | 0.696 | - | - | - | - | - |
|  | **12-17 yrs** | 0.826 | 0.729 | 0.711 | 0.703 | 0.837 | - | - | - | - | - |
|  | **Adults** | 0.537 | 0.553 | 0.471 | 0.728 | 0.698 | - | - | - | - | - |
| Morning 7  (chest clogged up) | **All ages** | 0.729 | 0.707 | 0.643 | 0.722 | 0.757 | 0.845 | - | - | - | - |
|  | **6-8 yrs** | 0.710 | 0.594 | 0.451 | 0.726 | 0.607 | 0.835 | - | - | - | - |
|  | **9-11 yrs** | 0.603 | 0.681 | 0.686 | 0.689 | 0.732 | 0.769 | - | - | - | - |
|  | **12-17 yrs** | 0.864 | 0.822 | 0.763 | 0.746 | 0.883 | 0.901 | - | - | - | - |
|  | **Adults** | 0.658 | 0.674 | 0.633 | 0.712 | 0.740 | 0.848 | - | - | - | - |
| Morning 8  (clear chest) | **All ages** | 0.695 | 0.749 | 0.612 | 0.658 | 0.697 | 0.704 | 0.733 | - | - | - |
|  | **6-8 yrs** | 0.643 | 0.753 | 0.484 | 0.631 | 0.699 | 0.733 | 0.616 | - | - | - |
|  | **9-11 yrs** | 0.638 | 0.758 | 0.475 | 0.635 | 0.484 | 0.523 | 0.675 | - | - | - |
|  | **12-17 yrs** | 0.810 | 0.794 | 0.792 | 0.645 | 0.832 | 0.795 | 0.835 | - | - | - |
|  | **Adults** | 0.637 | 0.680 | 0.582 | 0.769 | 0.658 | 0.679 | 0.760 | - | - | - |
| Morning 9  (clear throat) | **All ages** | 0.575 | 0.552 | 0.467 | 0.650 | 0.535 | 0.566 | 0.577 | 0.625 | - | - |
|  | **6-8 yrs** | 0.575 | 0.465 | 0.373 | 0.772 | 0.555 | 0.688 | 0.587 | 0.664 | - | - |
|  | **9-11 yrs** | 0.512 | 0.661 | 0.486 | 0.519 | 0.331 | 0.450 | 0.513 | 0.701 | - | - |
|  | **12-17 yrs** | 0.638 | 0.598 | 0.598 | 0.624 | 0.662 | 0.572 | 0.630 | 0.579 | - | - |
|  | **Adults** | 0.609 | 0.621 | 0.421 | 0.632 | 0.562 | 0.512 | 0.573 | 0.562 | - | - |
| Morning 10  (cough up mucus/goo) | **All ages** | 0.599 | 0.560 | 0.476 | 0.622 | 0.596 | 0.606 | 0.608 | 0.685 | 0.612 | - |
|  | **6-8 yrs** | 0.594 | 0.427 | 0.398 | 0.603 | 0.426 | 0.620 | 0.607 | 0.592 | 0.588 | - |
|  | **9-11 yrs** | 0.649 | 0.647 | 0.343 | 0.551 | 0.504 | 0.471 | 0.446 | 0.733 | 0.566 | - |
|  | **12-17 yrs** | 0.630 | 0.636 | 0.638 | 0.648 | 0.779 | 0.662 | 0.685 | 0.680 | 0.701 | - |
|  | **Adults** | 0.514 | 0.579 | 0.518 | 0.711 | 0.676 | 0.704 | 0.702 | 0.821 | 0.574 | - |
| *Cells shaded in green indicate items with correlations >0.8  *Cells shaded in yellow indicate items with correlations <0.4 | | | | | | | | | | | |

| Supplementary table 17. Inter-item Pearson correlations for the chest-related ePRO afternoon diary at Day 1 N=191; 6-8 years (n=42), 9-11 years (n=46), 12-17 years (n=55), adults (n=48) | | | | | | | | | | | | |
| --- | --- | --- | --- | --- | --- | --- | --- | --- | --- | --- | --- | --- |
|  |  | | Chest-related ePRO diary items | | | | | | | | | |
| Item | Age group | 1 | | 2 | 3 | 4 | 5 | 6 | 7 | 8 | 9 | 10 |
| **Afternoon 1 (difficulty breathing)** | **All ages** | - | | - | - | - | - | - | - | - | - | - |
|  | **6-8 yrs** | - | | - | - | - | - | - | - | - | - | - |
|  | **9-11 yrs** | - | | - | - | - | - | - | - | - | - | - |
|  | **12-17yrs** | - | | - | - | - | - | - | - | - | - | - |
|  | **Adults** | - | | - | - | - | - | - | - | - | - | - |
| **Afternoon 2  (chest tightness)** | **All ages** | 0.844 | | - | - | - | - | - | - | - | - | - |
|  | **6-8 yrs** | 0.854 | | - | - | - | - | - | - | - | - | - |
|  | **9-11 yrs** | 0.843 | | - | - | - | - | - | - | - | - | - |
|  | **12-17 yrs** | 0.857 | | - | - | - | - | - | - | - | - | - |
|  | **Adults** | 0.833 | | - | - | - | - | - | - | - | - | - |
| **Afternoon 3  (chest pain)** | **All ages** | 0.669 | | 0.690 | - | - | - | - | - | - | - | - |
|  | **6-8 yrs** | 0.748 | | 0.701 | - | - | - | - | - | - | - | - |
|  | **9-11 yrs** | 0.491 | | 0.590 | - | - | - | - | - | - | - | - |
|  | **12-17 yrs** | 0.800 | | 0.792 | - | - | - | - | - | - | - | - |
|  | **Adults** | 0.551 | | 0.624 | - | - | - | - | - | - | - | - |
| **Afternoon 4  (chest heaviness)** | **All ages** | 0.772 | | 0.807 | 0.697 | - | - | - | - | - | - | - |
|  | **6-8 yrs** | 0.861 | | 0.861 | 0.628 | - | - | - | - | - | - | - |
|  | **9-11 yrs** | 0.628 | | 0.733 | 0.687 | - | - | - | - | - | - | - |
|  | **12-17 yrs** | 0.811 | | 0.839 | 0.782 | - | - | - | - | - | - | - |
|  | **Adults** | 0.768 | | 0.765 | 0.702 | - | - | - | - | - | - | - |
| **Afternoon 5  (chest full of mucus)** | **All ages** | 0.638 | | 0.666 | 0.649 | 0.645 | - | - | - | - | - | - |
|  | **6-8 yrs** | 0.568 | | 0.674 | 0.526 | 0.567 | - | - | - | - | - | - |
|  | **9-11 yrs** | 0.513 | | 0.558 | 0.680 | 0.597 | - | - | - | - | - | - |
|  | **12-17 yrs** | 0.774 | | 0.722 | 0.722 | 0.721 | - | - | - | - | - | - |
|  | **Adults** | 0.689 | | 0.715 | 0.661 | 0.707 | - | - | - | - | - | - |
| **Afternoon 6  (chest stuffed up)** | **All ages** | 0.775 | | 0.771 | 0.722 | 0.740 | 0.760 | - | - | - | - | - |
|  | **6-8 yrs** | 0.791 | | 0.882 | 0.717 | 0.777 | 0.659 | - | - | - | - | - |
|  | **9-11 yrs** | 0.745 | | 0.718 | 0.604 | 0.540 | 0.667 | - | - | - | - | - |
|  | **12-17 yrs** | 0.805 | | 0.732 | 0.851 | 0.803 | 0.861 | - | - | - | - | - |
|  | **Adults** | 0.744 | | 0.730 | 0.705 | 0.792 | 0.905 | - | - | - | - | - |
| **Afternoon 7 (chest clogged up)** | **All ages** | 0.768 | | 0.761 | 0.745 | 0.787 | 0.812 | 0.823 | - | - | - | - |
|  | **6-8 yrs** | 0.871 | | 0.833 | 0.738 | 0.838 | 0.761 | 0.846 | - | - | - | - |
|  | **9-11 yrs** | 0.582 | | 0.648 | 0.736 | 0.673 | 0.777 | 0.684 | - | - | - | - |
|  | **12-17 yrs** | 0.842 | | 0.777 | 0.820 | 0.807 | 0.893 | 0.910 | - | - | - | - |
|  | **Adults** | 0.712 | | 0.773 | 0.648 | 0.811 | 0.792 | 0.837 | - | - | - | - |
| **Afternoon 8  (clear chest)** | **All ages** | 0.653 | | 0.710 | 0.610 | 0.669 | 0.764 | 0.717 | 0.728 | - | - | - |
|  | **6-8 yrs** | 0.526 | | 0.638 | 0.419 | 0.529 | 0.776 | 0.670 | 0.642 | - | - | - |
|  | **9-11 yrs** | 0.670 | | 0.704 | 0.585 | 0.628 | 0.647 | 0.578 | 0.640 | - | - | - |
|  | **12-17 yrs** | 0.816 | | 0.816 | 0.815 | 0.832 | 0.779 | 0.811 | 0.863 | - | - | - |
|  | **Adults** | 0.620 | | 0.679 | 0.583 | 0.669 | 0.847 | 0.821 | 0.735 | - | - | - |
| **Afternoon 9  (clear throat)** | **All ages** | 0.597 | | 0.628 | 0.585 | 0.615 | 0.567 | 0.679 | 0.609 | 0.683 | - | - |
|  | **6-8 yrs** | 0.678 | | 0.769 | 0.678 | 0.708 | 0.655 | 0.818 | 0.664 | 0.631 | - | - |
|  | **9-11 yrs** | 0.574 | | 0.564 | 0.563 | 0.493 | 0.526 | 0.633 | 0.536 | 0.744 | - | - |
|  | **12-17 yrs** | 0.575 | | 0.549 | 0.546 | 0.610 | 0.506 | 0.557 | 0.554 | 0.654 | - | - |
|  | **Adults** | 0.514 | | 0.630 | 0.494 | 0.609 | 0.583 | 0.649 | 0.735 | 0.758 | - | - |
| **Afternoon 10 (cough up mucus/goo)** | **All ages** | 0.633 | | 0.690 | 0.542 | 0.628 | 0.700 | 0.691 | 0.660 | 0.737 | 0.649 | - |
|  | **6-8 yrs** | 0.650 | | 0.717 | 0.485 | 0.627 | 0.645 | 0.648 | 0.649 | 0.725 | 0.620 | - |
|  | **9-11 yrs** | 0.645 | | 0.624 | 0.454 | 0.499 | 0.602 | 0.602 | 0.510 | 0.616 | 0.585 | - |
|  | **12-17 yrs** | 0.696 | | 0.729 | 0.656 | 0.680 | 0.761 | 0.767 | 0.742 | 0.755 | 0.694 | - |
|  | **Adults** | 0.542 | | 0.692 | 0.547 | 0.681 | 0.796 | 0.744 | 0.741 | 0.823 | 0.662 | - |
| *Cells shaded in green indicate items with correlations >0.8 | | | | | | | | | | | | |

## Exploratory factor analysis (EFA) and item reduction

| Supplementary table 18. Eigenvalues for the morning diary items in a single EFA | | | | | |
| --- | --- | --- | --- | --- | --- |
|  | Number of factors | | | | |
| Age group | 1 | 2 | 3 | 4 | 5 |
| Total sample | 7.61 | 0.55 | 0.39 | 0.35 | 0.31 |
| 6-11 year olds | 7.23 | 0.67 | 0.43 | 0.38 | 0.38 |
| 12+ year olds | 8.03 | 0.51 | 0.41 | 0.30 | 0.25 |

| Supplementary table 19. High modification indices for the morning diary items | | |
| --- | --- | --- |
| Age group | Items | Modification Indices |
| **Total sample** | 6 (stuffed up) and 7 (clogged up) | 36.97 |
| **12+ year olds** | 6 (stuffed up) and 7 (clogged up) | 20.90 |


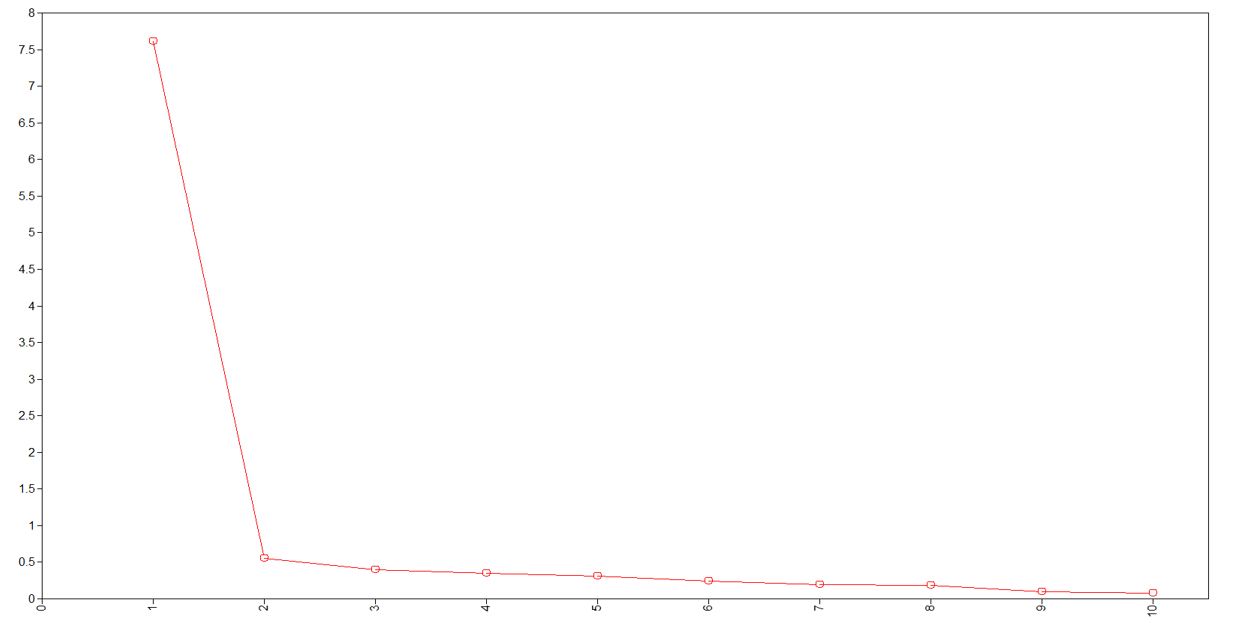


Supplementary figure 5. EFA scree plot for morning diary items in the total population

| Supplementary table 20. Eigenvalues for the afternoon diary items in a single EFA | | | | | |
| --- | --- | --- | --- | --- | --- |
|  | Number of factors | | | | |
| Age group | 1 | 2 | 3 | 4 | 5 |
| Total sample | 7.89 | 0.53 | 0.40 | 0.34 | 0.21 |
| 6-11 year olds | 7.60 | 0.62 | 0.53 | 0.35 | 0.29 |
| 12+ year olds | 8.23 | 0.57 | 0.37 | 0.26 | 0.18 |

| Supplementary table 21. High modification indices for the afternoon diary items | | |
| --- | --- | --- |
| Age group | Items | Modification Indices |
| **Total sample** | 1 (difficulty breathing) and 2 (chest tightness) | 28.90 |


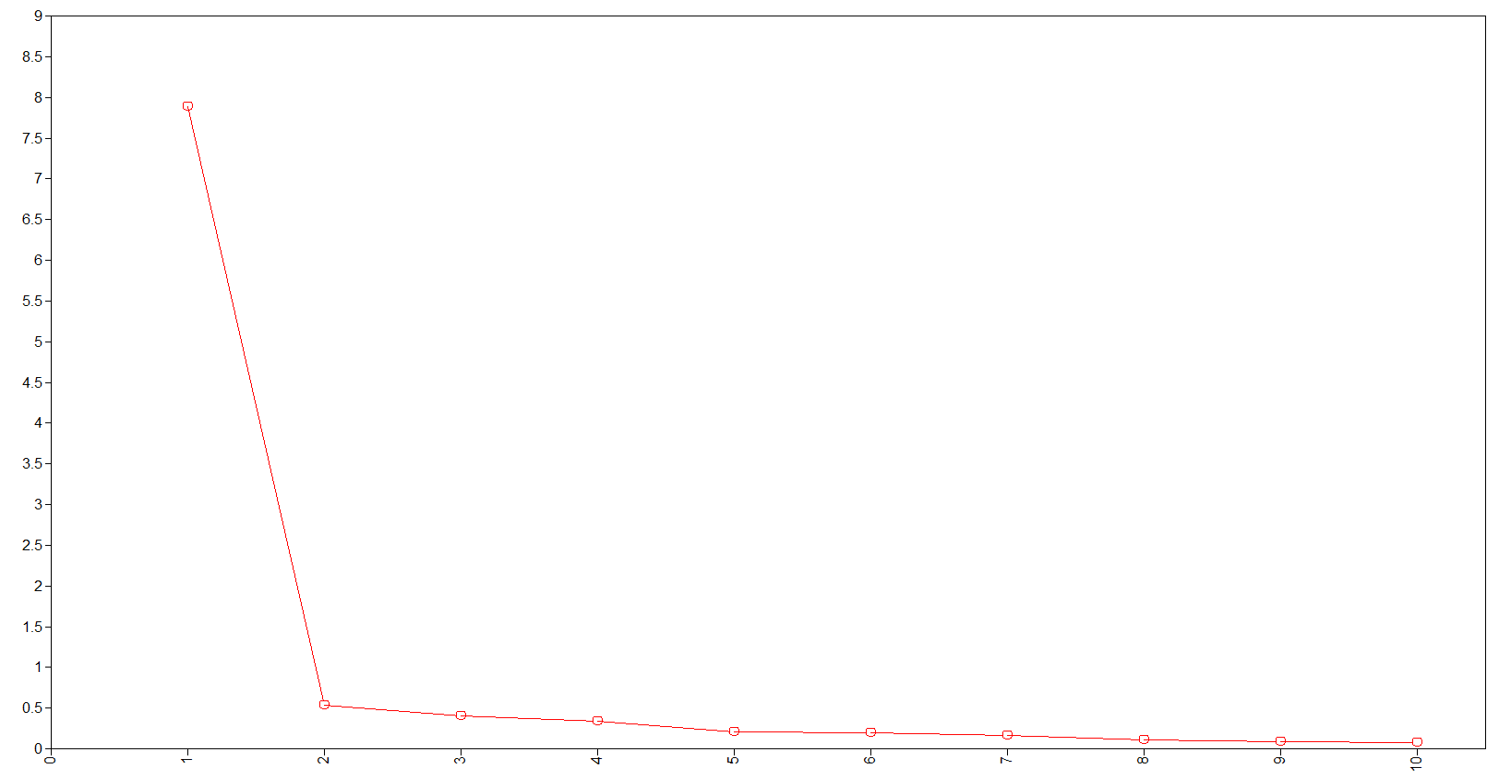


Supplementary figure 6. EFA scree plot for afternoon diary items in the total population

Items 3 (‘chest pain’) and 9 (‘clear throat’) did not group as closely with the other items in EFA analyses and, particularly item 9, provided relatively low information in IRT analyses. Uniform DIF according to age was also apparent for Item 9. Moreover, based on item content and the earlier qualitative findings, it was deemed that these two items were assessing concepts distal from the targeted underlying construct of chest congestion. Specifically, item 9 was the only item which asked about mucus in the throat rather than the chest. And in asking about chest pain, item 3 is arguably asked about too severe a concept.

Items 6 (‘chest stuffed up’) and 7 (‘chest clogged up’) were consistently very highly correlated suggesting redundancy. This was also evidenced by high MIs in the EFA and local dependence in IRT. The decision was taken to delete item 6 and retain item 7, on the basis that item 7 had a particularly strong IIC and provided the strongest reliability/information across the items.

Item 10 (‘cough up mucus/goo’) had one of the weaker IICs in the IRT analysis, suggesting it may not be very discriminative. However, after extensive discussions it was agreed to retain this item pending further examination of its performance using longitudinal data collected from an interventional study, as the item very closely assesses the expulsion of mucus, and so it may be important to retain. However, this item is flagged for careful consideration in future studies.

## Item response theory (IRT)

| Supplementary table 22. IRT S-X^2^ statistics for the chest-related ePRO diary morning items for the total sample (N=195), the 6-11 year old age group (N=89) and the 12+ year old age group (N=106) | | | | |
| --- | --- | --- | --- | --- |
| Item | Age group | S-X^2^ | Degrees of freedom | P-value |
| Morning 1 (difficulty breathing) | Total sample | 32.45 | 30 | 0.3462 |
|  | 6-11 age group | 20.05 | 14 | 0.1281 |
|  | 12+ age group | 13.99 | 11 | 0.2332 |
| Morning 2 (chest tightness) | Total sample | 44.16 | 28 | 0.0267 |
|  | 6-11 age group | 22.68 | 14 | 0.0656 |
|  | 12+ age group | 19.13 | 12 | 0.0851 |
| Morning 3 (chest pain) | Total sample | 35.05 | 39 | 0.6513 |
|  | 6-11 age group | 17.41 | 22 | 0.7409 |
|  | 12+ age group | 16.34 | 17 | 0.501 |
| Morning 4 (chest heaviness) | Total sample | 25.06 | 26 | 0.5171 |
|  | 6-11 age group | 14.72 | 13 | 0.3268 |
|  | 12+ age group | 26.03 | 14 | 0.0256 |
| Morning 5 (chest full of mucus) | Total sample | 41.74 | 30 | 0.0751 |
|  | 6-11 age group | 21.08 | 14 | 0.0994 |
|  | 12+ age group | 16.45 | 12 | 0.1711 |
| Morning 6 (chest stuffed up) | Total sample | 35.39 | 28 | 0.1585 |
|  | 6-11 age group | 22.24 | 16 | 0.1351 |
|  | 12+ age group | 18.3 | 10 | 0.0499 |
| Morning 7 (chest clogged up) | Total sample | 34.53 | 24 | 0.0755 |
|  | 6-11 age group | 20.11 | 13 | 0.0922 |
|  | 12+ age group | 14 | 8 | 0.0815 |
| Morning 8 (clear chest) | Total sample | 37.89 | 34 | 0.2954 |
|  | 6-11 age group | 16.61 | 15 | 0.3443 |
|  | 12+ age group | 27.43 | 17 | 0.0519 |
| Morning 9 (clear throat) | Total sample | 48.77 | 40 | 0.1608 |
|  | 6-11 age group | 23.03 | 18 | 0.189 |
|  | 12+ age group | 21.94 | 20 | 0.3458 |
| Morning 10 (cough up mucus/goo) | Total sample | 64.68 | 39 | 0.006 |
|  | 6-11 age group | 25.44 | 19 | 0.1462 |
|  | 12+ age group | 30.75 | 17 | 0.0214 |

*Cells shaded in yellow indicate p<0.01)*

| Supplementary table 23. IRT S-X2 statistics for the chest-related ePRO diary afternoon items for the total sample (N=195), the 6-11 year old age group (N=89) and the 12+ year old age group (N=106) | | | | |
| --- | --- | --- | --- | --- |
| Item | Age group | S-X^2^ | Degrees of freedom | P-value |
| Afternoon 1 (difficulty breathing) | Total sample | 49.2 | 33 | 0.0345 |
|  | 6-11 age group | 30.07 | 11 | 0.0015 |
|  | 12+ age group | 40.27 | 16 | 0.0007 |
| Afternoon 2 (chest tightness) | Total sample | 25.02 | 27 | 0.5744 |
|  | 6-11 age group | 15.51 | 11 | 0.1597 |
|  | 12+ age group | 15.14 | 16 | 0.5158 |
| Afternoon 3 (chest pain) | Total sample | 36.16 | 35 | 0.4155 |
|  | 6-11 age group | 25.97 | 15 | 0.0382 |
|  | 12+ age group | 24.27 | 11 | 0.0116 |
| Morning 4 (chest heaviness) | Total sample | 26.32 | 28 | 0.5568 |
|  | 6-11 age group | 18.33 | 13 | 0.145 |
|  | 12+ age group | 19.15 | 12 | 0.0847 |
| Afternoon 5 (chest full of mucus) | Total sample | 34.59 | 31 | 0.2994 |
|  | 6-11 age group | 23.13 | 16 | 0.1099 |
|  | 12+ age group | 26.43 | 13 | 0.0148 |
| Afternoon 6 (chest stuffed up) | Total sample | 29.2 | 25 | 0.2549 |
|  | 6-11 age group | 19.74 | 10 | 0.0317 |
|  | 12+ age group | 15.14 | 6 | 0.0192 |
| Afternoon 7 (chest clogged up) | Total sample | 25.85 | 26 | 0.4729 |
|  | 6-11 age group | 15.52 | 11 | 0.1593 |
|  | 12+ age group | 18.26 | 9 | 0.0321 |
| Afternoon 8 (clear chest) | Total sample | 39.38 | 33 | 0.2054 |
|  | 6-11 age group | 26.99 | 13 | 0.0124 |
|  | 12+ age group | 25.97 | 12 | 0.0108 |
| Afternoon 9 (clear throat) | Total sample | 39.87 | 39 | 0.4328 |
|  | 6-11 age group | 20.12 | 18 | 0.3253 |
|  | 12+ age group | 25.43 | 19 | 0.1466 |
| Afternoon 10 (cough up mucus/goo) | Total sample | 46.84 | 36 | 0.1064 |
|  | 6-11 age group | 27.47 | 11 | 0.0039 |
|  | 12+ age group | 28.03 | 15 | 0.0213 |

*Cells shaded in yellow indicate p<0.01)*

*
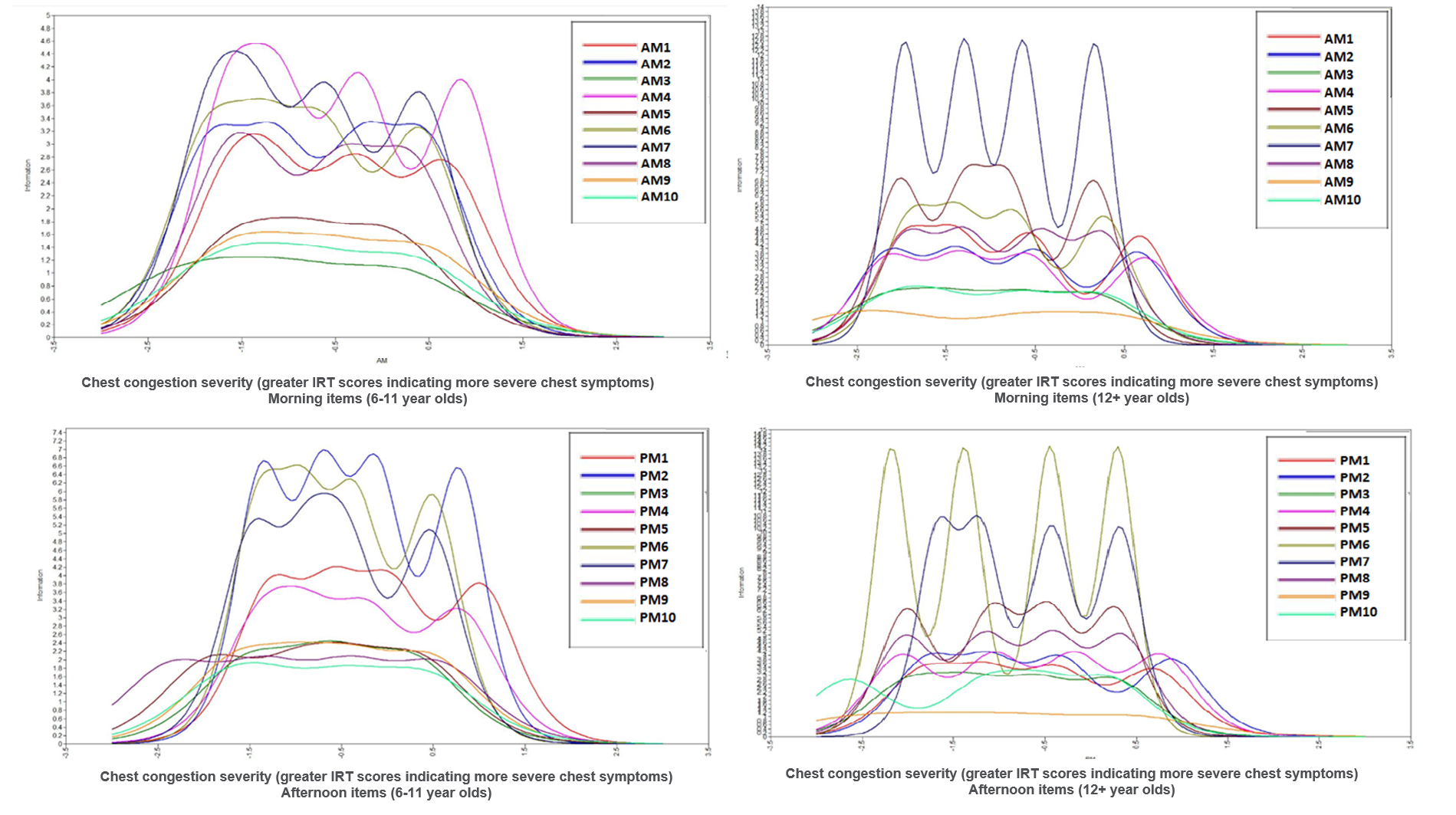
*

Supplementary figure 7. IICs for AM and PM items in age subgroups

## Internal consistency reliability

| Supplementary table 24. Internal consistency and reliability for total diary items (Morning & afternoon) Day 1 | | | | | |
| --- | --- | --- | --- | --- | --- |
| Diary items | Total sample (N=191*) | 6–8-year-olds (N=42) | 9–11-year-olds (N=46) | 12–17-year-olds (N=55) | Adults (N=48) |
| Overall internal Consistency | **0.964** | **0.957** | **0.957** | **0.975** | **0.960** |
| Alpha coefficient with each item deleted: | |  |  |  |  |
| Morning 1 (Difficulty breathing) | 0.961 | 0.955 | 0.952 | 0.973 | 0.959 |
| Morning 2 (chest tightness) | 0.961 | 0.956 | 0.952 | 0.973 | 0.956 |
| Morning 4 (chest heaviness) | 0.961 | 0.952 | 0.952 | 0.975 | 0.958 |
| Morning 5 (chest full of mucus) | 0.963 | 0.958 | 0.956 | 0.973 | 0.956 |
| Morning 7 (chest clogged up) | 0.961 | 0.954 | 0.953 | 0.972 | 0.958 |
| Morning 8 (clear chest) | 0.961 | 0.953 | 0.955 | 0.973 | 0.958 |
| Morning 10 (cough up mucus) | 0.963 | 0.956 | 0.957 | 0.975 | 0.958 |
| Afternoon 1 (Difficulty breathing) | 0.961 | 0.951 | 0.953 | 0.972 | 0.958 |
| Afternoon 2 (chest tightness) | 0.960 | 0.952 | 0.952 | 0.973 | 0.957 |
| Afternoon 4 (chest heaviness) | 0.961 | 0.953 | 0.954 | 0.974 | 0.956 |
| Afternoon 5 (chest full of mucus) | 0.961 | 0.955 | 0.954 | 0.973 | 0.955 |
| Afternoon 7 (chest clogged up) | 0.960 | 0.951 | 0.953 | 0.972 | 0.957 |
| Afternoon 8 (clear chest) | 0.962 | 0.956 | 0.953 | 0.973 | 0.957 |
| Afternoon 10 (cough up mucus) | 0.962 | 0.953 | 0.955 | 0.975 | 0.957 |
| **N=191 in the analysis does not equal the full study sample due to missing completions from four participants.* | | | | | |

## Exit interview findings

| **Supplementary table 25. Levels of understanding for individual participants and age groups** | | | | | | | | | | | | |
| --- | --- | --- | --- | --- | --- | --- | --- | --- | --- | --- | --- | --- |
| Age group | Participant ID | ePRO item | | | | | | | | | | Total understood in age group (%) |
|  |  | 1 | 2 | 3 | 4 | 5 | 6 | 7 | 8 | 9 | 10 |  |
| **6-8 years** | 006-022-F-6* | - | u | u | 🗶 | 🗶 | u | 🗶 | u | 🗶 | U | 60 |
|  | 006-034-F-7* | u | u | ✓ | 🗶 | 🗶 | ✓ | ✓ | ✓ | ✓ | - |  |
|  | 012-009-F-7* | ✓ | ✓ | u | 🗶 | ✓ | ✓ | ✓ | ✓ | ✓ | ✓ |  |
|  | 006-018-F-8* | ✓ | ✓ | ✓ | ✓ | - | ✓ | ✓ | ✓ | ✓ | u |  |
|  | 012-012-F-8* | ✓ | ✓ | ✓ | 🗶 | ✓ | ✓ | ✓ | ✓ | - | u |  |
|  | 006-043-F-7* | ✓ | - | - | u | 🗶 | ✓ | - | - | - | - |  |
| **% understood item** | | 80 | 60 | 60 | 17 | 40 | 83 | 80 | 80 | 75 | 25 |  |
| **9-11 years** | 012-028-M-9 | - | 🗶 | ✓ | ✓ | ✓ | - | ✓ | ✓ | - | - | 70 |
|  | 006-035-F-9 | ✓ | 🗶 | ✓ | - | u | u | u | u | ✓ | u |  |
|  | 006-021-M-9 | ✓ | u | ✓ | - | ✓ | u | ✓ | ✓ | ✓ | ✓ |  |
|  | 005-001-F-10 | ✓ | 🗶 | ✓ | ✓ | 🗶 | ✓ | ✓ | ✓ | 🗶 | ✓ |  |
|  | 010-013-M-10 | ✓ | ✓ | ✓ | ✓ | ✓ | ✓ | ✓ | ✓ | ✓ | ✓ |  |
|  | 007-014-F-11 | ✓ | ✓ | ✓ | ✓ | 🗶 | ✓ | ✓ | ✓ | 🗶 | ✓ |  |
|  | 012-013-M-11 | ✓ | 🗶 | u | 🗶 | ✓ | 🗶 | 🗶 | ✓ | ✓ | ✓ |  |
|  | 012-022-F-11 | - | ✓ | - | ✓ | ✓ | - | ✓ | ✓ | - | u |  |
| **% understood item** | | 100 | 38 | 86 | 83 | 63 | 50 | 75 | 88 | 67 | 71 |  |
| **12-17 years** | 012-011-F-12 | ✓ | ✓ | ✓ | ✓ | ✓ | ✓ | ✓ | ✓ | u | u | 78 |
|  | 012-014-M-12 | ✓ | ✓ | ✓ | ✓ | 🗶 | ✓ | ✓ | ✓ | ✓ | ✓ |  |
|  | 002-014-F-14 | ✓ | ✓ | ✓ | u | - | 🗶 | ✓ | ✓ | ✓ | ✓ |  |
|  | 006-019-F-14 | ✓ | ✓ | 🗶 | 🗶 | ✓ | ✓ | ✓ | ✓ | ✓ | 🗶 |  |
|  | 006-033-F-14 | ✓ | ✓ | ✓ | 🗶 | ✓ | u | u | ✓ | ✓ | ✓ |  |
|  | 009-006-M-15 | ✓ | ✓ | ✓ | 🗶 | ✓ | ✓ | ✓ | ✓ | - | ✓ |  |
|  | 014-011-F-17 | ✓ | ✓ | ✓ | ✓ | ✓ | ✓ | ✓ | ✓ | ✓ | ✓ |  |
| **% understood item** | | 100 | 100 | 86 | 29 | 83 | 71 | 86 | 100 | 83 | 71 |  |
| **18+ years** | 006-001-F-23 | ✓ | ✓ | ✓ | ✓ | ✓ | ✓ | ✓ | ✓ | ✓ | ✓ | 91 |
|  | 006-011-F-25 | ✓ | ✓ | ✓ | ✓ | ✓ | u | ✓ | ✓ | ✓ | ✓ |  |
|  | 003-011-F-27 | ✓ | ✓ | ✓ | ✓ | ✓ | ✓ | ✓ | u | ✓ | ✓ |  |
|  | 007-003-F-32 | ✓ | ✓ | ✓ | ✓ | 🗶 | u | u | - | - | - |  |
|  | 005-005-F-36 | ✓ | ✓ | ✓ | - | - | - | - | - | - | - |  |
|  | 005-004-F-38 | ✓ | ✓ | ✓ | 🗶 | - | - | - | - | - | - |  |
|  | 006-003-F-43 | ✓ | ✓ | ✓ | ✓ | ✓ | u | ✓ | ✓ | ✓ | ✓ |  |
|  | 004-010-M-64 | ✓ | ✓ | ✓ | ✓ | ✓ | ✓ | ✓ | ✓ | ✓ | ✓ |  |
|  | 004-011-F-67 | ✓ | ✓ | ✓ | ✓ | ✓ | ✓ | ✓ | ✓ | ✓ | ✓ |  |
| **% understood item** | | 100 | 100 | 100 | 88 | 86 | 57 | 86 | 83 | 100 | 100 |  |
| ✓: Understood 🗶: Not understood u: Unclear -: Not asked | | | | | | | | | | | | |
